# Coumarin Derivatives as Anticancer Agents: Mechanistic Landscape with an Emphasis on Breast Cancer

**DOI:** 10.3390/molecules30214167

**Published:** 2025-10-23

**Authors:** Veda B. Hacholli, Shubha M. R., Prabhanajan B. H., Lavanya M., Pramod S., Abhishek Kumar, Łukasz Szeleszczuk, Marcin Gackowski

**Affiliations:** 1 Department of Pharmaceutical Chemistry, Faculty of Pharmacy, Nitte College of Pharmaceutical Sciences, Nitte (Deemed to be University), Bengaluru 560064, KA, India; vedab.hacholli@nitte.edu.in (V.B.H.); shubha.mr@nitte.edu.in (S.M.R.); lavanyam152003@gmail.com (L.M.); pramodraj082003@gmail.com (P.S.); 2 ITAAN PHARMA PVT. Ltd., Plot No S-9, Genome Valley Phase III, Biotech Park, Karkapatla 5022812, TG, India; p.hacholli1998@gmail.com; 3 Department of Pharmaceutical Chemistry, NGSM Institute of Pharmaceutical Sciences (NGSMIPS), Nitte (Deemed to be University), Mangalore 560064, KA, India; 4 Department of Organic and Physical Chemistry, Faculty of Pharmacy, Medical University of Warsaw, Banacha 1 Street, PL-02093 Warsaw, Poland; lukasz.szeleszczuk@wum.edu.pl; 5 Department of Toxicology and Bromatology, Faculty of Pharmacy, L. Rydygier Collegium Medicum in Bydgoszcz, Nicolaus Copernicus University in Torun, A. Jurasza 2 Street, PL-85089 Bydgoszcz, Poland

**Keywords:** coumarin, furocoumarin, breast cancer, VEGFR-2, aromatase, apoptosis, PI3K/Akt/mTOR, multidrug resistance

## Abstract

Coumarin derivatives constitute a versatile small-molecule chemotype with broad anticancer potential. This narrative review synthesizes recent in vitro and in vivo evidence on coumarin-based scaffolds, emphasizing breast cancer and covering lung, prostate, and colorectal models. We summarize major mechanisms of action—including induction of apoptosis (caspase activation and BAX/BCL-2 balance), modulation of PI3K/Akt/mTOR signaling, inhibition of angiogenesis (VEGFR-2), interference with estrogen biosynthesis (aromatase/ER axis), chaperone targeting (Hsp90), and attenuation of multidrug resistance (efflux pumps/autophagy)—and highlight representative chemotypes (e.g., benzimidazole, triazole, furocoumarins, topoisomerase- and CDK-oriented hybrids). Where available, we contrast potency and selectivity across models (e.g., MCF-7 vs. MDA-MB-231; A549; PC-3; colon lines) and discuss structure–activity trends linking substituent patterns (heteroaryl linkers, judicious halogenation, polar handles) to pathway engagement. We also delineate translational gaps limiting clinical progress—selectivity versus non-malignant cells, incomplete pharmacokinetic and safety characterization, and limited validation beyond xenografts. Finally, we outline priorities for preclinical optimization: biology-aligned target selection with biomarkers, resistance-aware combinations (e.g., PI3K/mTOR ± autophagy modulation; MDR mitigation), and early integration of ADME/tox and PK/PD to confirm on-target exposure. Collectively, the evidence supports coumarins as adaptable, multi-target anticancer leads, particularly promising in hormone-dependent breast cancer while remaining relevant to other tumor types.

## 1. Introduction

Cancer has been identified as a leading cause of mortality globally, with projections by the World Health Organization indicating that annual cancer-related deaths could reach approximately 13.2 million by 2030 [1]. Although numerous chemotherapeutic agents have been developed to arrest aberrant cell proliferation in various cancers, their clinical application is significantly constrained by the serious adverse effects they induce. Cancer is ranked as the second most common cause of death globally, preceded only by cardiovascular disease, and is recognized as a significant threat to public health. Cancer is capable of invading or metastasizing to nearly all regions of the body [2]. The escalating morbidity and mortality rates associated with cancer have generated a demanding need for the development of novel anticancer therapeutics. Although some standards have been determined for cancer therapy, various approaches and treatments are applied to a specific type of cancer. Biological therapies, such as radiotherapy, chemotherapy, surgery, immunotherapy, hormone therapy, targeted therapies, and gene therapy, can be used alone or in combination in cancer therapy. However, these methods, known as the gold standard, have advantages and disadvantages [3]. Despite the discovery of many chemotherapeutic drugs (Adriamycin, Cisplatin, Camptothecin, Vinblastin, Mercaptopurine, etc.) that inhibit uncontrolled cell division process for the treatment of different types of cancer, serious side effects of these drugs on hematopoietic system, bone marrow and gastrointestinal epithelial cells and hair follicles are a crucial disadvantage In addition, multidrug resistance (MDR) is another critical problem in anticancer treatment. Due to cytotoxicity and drug resistance in existing chemotherapeutic agents, many investigations are being conducted to discover and develop effective anticancer drugs. Previous studies showed that many compounds obtained from natural resources can be used as preventive and therapeutic agents in cancer therapy [4]. These compounds have been shown to increase the effectiveness and tolerance of chemotherapeutic agents when combined with chemotherapy or alone in various types of cancers. Researchers have recently focused on the anticancer activity of coumarin and coumarin-derived natural compounds among many phytochemicals due to their high biological activity and low toxicity [5]. Coumarins are commonly used primarily in the treatment of prostate cancer, renal cell carcinoma, and leukemia, and they can also counteract the side effects caused by radiotherapy. It revealed that combining coumarin and troxerutin positively impacts treating the malignancy of head and neck radiotherapy. Both coumarin and coumarin derivatives are promising compounds as potential inhibitors of cell proliferation in various carcinoma cell lines [6].

We conducted a focused narrative search across PubMed, Scopus, and Web of Science using combinations of “coumarin*” AND (anticancer OR antitumor OR oncology) together with mechanism-related terms (e.g., “VEGFR-2”, “EGFR”, “PI3K”, “PARP”, “Hsp90”, “P-gp”). We prioritized peer-reviewed primary studies and recent medicinal-chemistry reports that included in vitro and/or in vivo preclinical models in breast, lung, prostate, and colorectal cancers; we excluded non-anticancer indications, non-English items, reviews (unless used for backward/forward snowballing), conference abstracts, and duplicates. Titles/abstracts were screened with complete-text verification where needed; the most comprehensive or recent dataset was retained when multiple reports addressed the same chemotype. For each study, we extracted chemotype/class, target(s), model(s), potency (IC50/Ki), dominant phenotype, and notable selectivity/PK observations. As this narrative review aimed at mapping design trends, we did not perform a formal risk-of-bias assessment or meta-analysis.

This study makes several significant contributions to the field of cancer research. It provides a comprehensive review of the anti-cancer potential of coumarin derivatives, explicitly focusing on breast cancer [7]. The study demonstrates these compounds’ molecularly targeted therapeutic effects, including the induction of apoptosis, suppression of angiogenesis, and inhibition of metastases [8]. Furthermore, it provides valuable insights into the molecular mechanisms influenced by coumarin derivatives, highlighting their impact on various signalling pathways involved in regulating cell growth, survival, and programmed cell death. This research establishes a foundation for future clinical trials and optimization strategies, paving the way for their translational potential in cancer treatment [3]. This investigation underscores the promise of coumarin derivatives as innovative therapeutic agents, addressing the ethical need for safer and more effective treatment strategies against breast cancer [9].

## 2. Coumarin Characteristics

The term “coumarin” derives from the French coumarou—the name for the tonka bean—and ultimately from indigenous Cariban (Galibi) and Old Tupi (kumarú), which also inspired the former genus name Coumarouna. Coumarin (benzopyran-2-one; chromen-2-one) is a naturally occurring aromatic lactone composed of a fused benzene and α-pyrone ring. It is found in tonka beans and sweet clover, among other plants, and has been associated with diverse pharmacological properties [10,11]. Beyond its natural occurrence, the coumarin scaffold underpins numerous bioactive derivatives, including widely used 4-hydroxycoumarin anticoagulants (e.g., warfarin), in which the coumarin core is retained as a key structural motif. Structurally, coumarin is classified as an aromatic lactone featuring a conjugated system contributing to its characteristic odor and reactivity [11].

**Figure 1:**
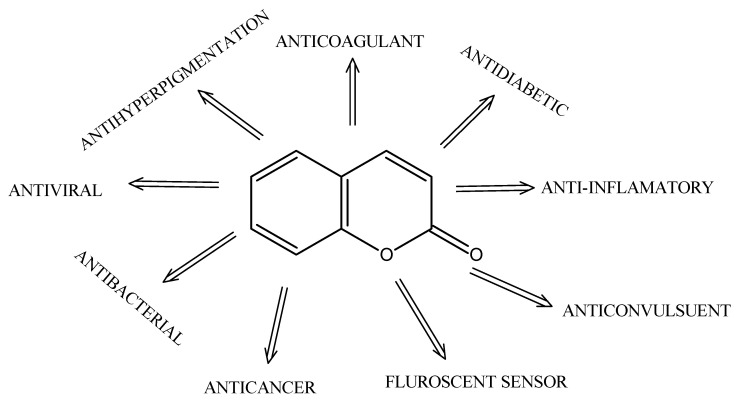
Therapeutic and diagnostic applications associated with coumarin derivatives.

More than 300 coumarin derivatives have been identified as secondary metabolites in plant seeds, roots, and leaves, spanning a broad range of biological and potential therapeutic activities [12]. Representative pharmacological themes include antioxidant, anti-inflammatory, antimicrobial, and anticancer effects, which continue to motivate medicinal-chemistry exploration of this privileged scaffold [10,12] (Figure 1).

## 3. Mechanistic Exploration of Coumarin Derivatives as Promising Anticancer Agents

Coumarins constitute a privileged scaffold with a distinct structural motif linked to diverse biological functions and pharmacological activities. They can be obtained either by de novo synthesis or by isolation from plant sources. This review focuses on anticancer mechanisms reported for synthetic and naturally derived coumarins over the past decade [13]. Representative examples illustrate the breadth of target engagement: isopentenyloxycoumarins suppress angiogenesis by downregulating CCL2 chemokine levels; Ferulin C, a potent colchicine-binding microtubule disruptor, inhibits breast-cancer cell proliferation and metastasis via PAK1/PAK2-dependent signaling; triphenylethylene–coumarin hybrid trimers display marked growth inhibition in HeLa, A549, K562 and MCF-7 cell lines; platinum(IV) complexes of 4-hydroxycoumarin trigger apoptosis in SKOV-3 cells through enhanced caspase-3 and caspase-9 activation and exert strong genotoxic effects in tumor models; 3-benzylcoumarin derivatives with a seco-B-ring architecture induce apoptosis by modulating the PI3K/Akt/mTOR axis [14]. Additional mechanistic avenues include inhibition of MDR-associated efflux transporters by sesquiterpene coumarins and aromatase suppression by imidazolyl-substituted coumarins, which are relevant in estrogen-dependent breast cancer.

**Figure 2:**
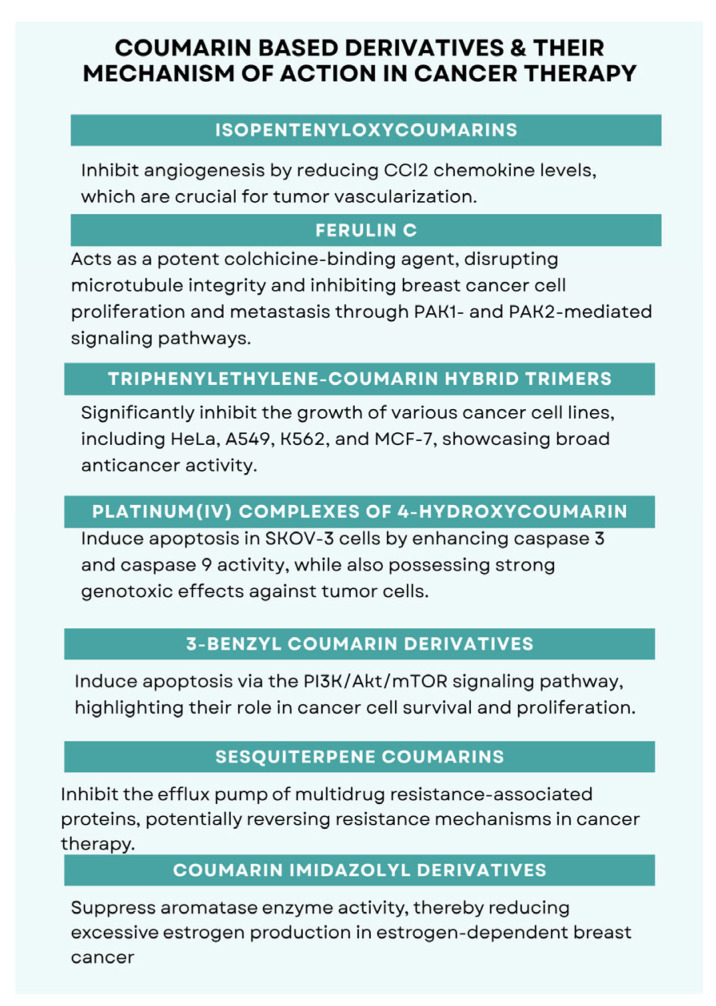
Mechanistic landscape of representative coumarin-based derivatives in cancer models (anti-angiogenic activity, proliferation blockade, apoptosis induction, microtubule disruption, and estrogen-biosynthesis suppression).

These findings position coumarin-based derivatives as multi-target anticancer agents capable of modulating apoptosis, cell-cycle control, microtubule dynamics, angiogenesis/chemokine signaling, drug-resistance mechanisms, and estrogen biosynthesis. Figure 2 summarizes these mechanisms for the above compound classes. From a structural perspective, Section 9 consolidates the central substitution motifs (C-4 aryl/benzyl, C-7 heteroaryl/alkoxy, linker length/polarity, halogenation) alongside their prevalent targets and phenotypes.

From a translational perspective, the orthogonality of these mechanisms suggests opportunities for rational combinations (e.g., microtubule disruption alongside PI3K/Akt/mTOR modulation) and scaffold-guided selectivity optimization. Structure–activity observations across cell models (e.g., MCF-7 vs. MDA-MB-231, HeLa, A549) indicate that subtle substituent patterns can bias pathway engagement and apoptotic readouts (caspase activation, BAX/BCL-2 balance). Equally important are resistance-aware designs that address MDR transporters while maintaining acceptable effects on non-malignant cells; selectivity indices and early ADME/tox profiling remain critical benchmarks for progression.

Several coumarin chemotypes demonstrate parallel pathway engagement. For example, coumarin–benzimidazole hybrids suppress PI3K/Akt/mTOR signaling with caspase-dependent apoptosis, while natural/furanocoumarin scaffolds from Citrus trifoliata inhibit P-glycoprotein and resensitize multidrug-resistant cells to doxorubicin—illustrating how signal attenuation and efflux modulation can be co-opted within a single coumarin-centered design strategy [15,16]. In lung models, NO-releasing coumarin–furoxans induce apoptosis alongside cytoprotective autophagy downstream of Akt/mTOR, motivating combination with autophagy blockers when that phenotype is observed [17].

**Figure 3:**
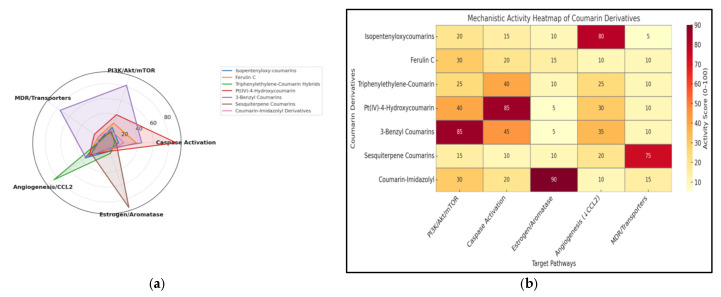
Comparative pathway profiling of coumarin derivatives. (**a**) Radar plot illustrating pathway selectivity. (**b**) Heatmap showing relative activity scores across caspase, PI3K/Akt/mTOR, MDR, angiogenesis/CCL2, and estrogen/aromatase axes.

To complement the qualitative overview in Figure 2, Figure 3 presents a pathway-focused comparison across key axes—caspase activation, PI3K/Akt/mTOR signaling, MDR transporters, angiogenesis/CCL2, and estrogen/aromatase—using a radar plot and a heatmap to visualize relative activity patterns [18,19]. Briefly, pathway-specific readouts extracted from the cited studies (enzymatic percent inhibition or IC50; cellular markers such as cleaved caspase-3, p-Akt, aromatase activity, CCL2) were normalized to a 0–100 relative-activity scale: strong (≥70% inhibition or IC50 ≤ 5 µM, or ≥2-fold pro-apoptotic change/≥2-fold decrease in p-Akt/aromatase/CCL2) = 100; moderate (30–69% inhibition or 5–20 µM, or 1.3–1.9-fold change) = 50; weak (<30% inhibition or >20 µM, or <1.3-fold change) = 0. Panel (a) shows these values with 20-unit ticks on the radar; panel (b) displays the same 0–100 scale as a heatmap. Radar traces reflect per-compound maxima per axis; the heatmap summarizes per-axis medians across exemplars. These plots are comparative visualizations rather than a meta-analysis.

## 4. Coumarin-Based Derivatives for Targeted Prostate Cancer Therapy

Within prostate cancer (PCa), coumarin derivatives have been explored as modulators of cell-cycle progression, apoptotic signaling, cellular motility, and pro-survival pathways. Selected representatives are summarized below. For a concise map of motifs and targets pertinent to this section, see Section 9, Table 1.

### 4.1. Esculetin (6,7-Dihydroxycoumarin): Induction of Apoptosis and G1 Arrest in Human Prostate Cancer Cells

Across PCa cell lines PC3, DU145, and LNCaP, esculetin reduced cell viability in a dose- and time-dependent manner [20]. Consistent with cytostatic activity, cell-cycle analyses demonstrated G1-phase arrest accompanied by upregulation of p53, p21 (Cip1), and p27 (Kip1), together with downregulation of CDK2, CDK4, and cyclin D [19]. Esculetin also induced apoptosis (Annexin V/PI), associated with increased cytochrome c and PTEN expression and decreased Akt phosphorylation, findings compatible with activation of intrinsic—and possibly extrinsic—apoptotic pathways [21]. In motility assays, esculetin inhibited cell migration, particularly at higher concentrations, potentially via suppression of Akt signaling and downstream EMT regulators, including Snail and Twist [22].

Overall, esculetin exhibits antiproliferative, pro-apoptotic, and anti-migratory effects in both androgen-dependent (LNCaP) and androgen-independent (PC3, DU145) PCa models In Vitro. By modulating the PTEN/Akt axis alongside key cell-cycle regulators, esculetin emerges as a therapeutic candidate warranting further preclinical evaluation to establish efficacy, selectivity, and translational potential [23].

### 4.2. Benzylidene Coumarin Hybrids as Targeted Anti-Prostate Cancer Agents: Dual Inhibition of EGFR and PI3Kβ

**Figure 4:**
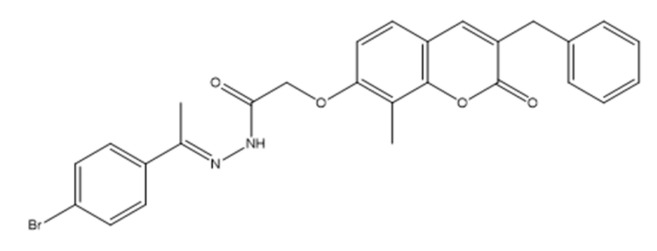
The chemical structure of compound **1**.

Recent work has identified newly developed coumarin derivatives as promising candidates for targeted cancer therapy. Among these, compound **1**—(E)-2-((3-benzyl-8-methyl-2-oxo-2H-chromen-7-yl)oxy)-N’-(1-(4-bromophenyl)ethylidene)acetohydrazide (Figure 4)—showed notable cytotoxicity against human prostate cancer PC-3 and triple-negative breast cancer MDA-MB-231 cell lines, with IC_50_ = 3.56 µM and 8.5 µM, respectively, reportedly exceeding the potency of erlotinib (quinazoline-based EGFR tyrosine-kinase inhibitor; non-coumarin) under comparable conditions [24].

Mechanistically, the activity of compound **1** has been attributed to dual inhibition of EGFR (PDB: 1M17) and PI3Kβ (PDB: 2Y3A), with biochemical IC_50_ values of 0.1812 µM and 0.2612 µM, respectively [25]. Molecular docking supported a strong binding affinity within the active sites of both kinases, consistent with the observed biochemical inhibition.

In cellular systems, treatment of PC-3 cells led to disruption of the EGFR/PI3K/Akt/mTOR cascade, reflected by decreased expression/phosphorylation levels of Akt and mTOR, indicating effective pathway suppression [26]. Cell-cycle analysis demonstrated an S-phase block, suggesting interference with DNA synthesis and progression through the S phase. Apoptosis was induced Via intrinsic and extrinsic pathways, as evidenced by elevated caspase-3, caspase-8, p53, and BAX levels alongside reduced BCL-2.

Collectively, these data point to a multifaceted mode of action in which compound **1** simultaneously impairs proliferative signaling and promotes programmed cell death. Coumarin-based molecular frameworks—exemplified by this benzylidene coumarin hybrid—thus represent promising lead structures for further optimization toward anticancer agents capable of multi-target engagement and potential resistance mitigation [27].

### 4.3. Coumarin–Benzimidazole Compounds as Emerging Anticancer Agents in Prostate Cancer Therapy

**Figure 5:**
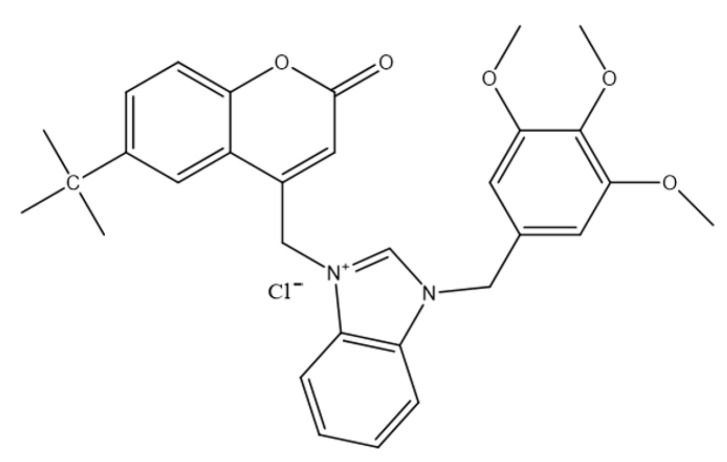
Chemical structure of compound **2**.

A recent study reported a series of coumarin–benzimidazole hybrids designed to merge two pharmacologically active scaffolds with established anticancer credentials. Six 6-substituted-4-chloromethylene coumarins were prepared and coupled with N-benzylbenzimidazole derivatives to afford sixteen coumarin–benzimidazolium chlorides [4]. Cytotoxicity was assessed against PC-3 human prostate cancer cells using the MTT viability assay at 1, 10, and 100 μM. All compounds reduced cell viability at the highest concentration, and several retained notable activity even at 1 μM, indicating promising potency within the low-micromolar range. Compound **2**—3-((6-(tert-butyl)-2-oxo-2H-chromen-4-yl)methyl)-1-(3,4,5-trimethoxybenzyl)-1H-benzo[d]imidazol-3-ium (Figure 5)—emerged as the most active candidate in this panel [4].

Although the exact mechanism remains to be fully defined, the activity profile of these hybrids is consistent with pathways commonly associated with coumarin and benzimidazole derivatives, including mitochondrial dysfunction leading to apoptosis, DNA intercalation, and inhibition of tubulin polymerization [28]. While overall potency was lower than docetaxel, the combination of low-micromolar cytotoxicity and a potentially favorable toxicity outlook supports their consideration as lead structures for optimization. Notably, pentamethylbenzyl-substituted derivatives were less active, suggesting that benzyl substitution patterns critically modulate efficacy and warrant systematic exploration in follow-up libraries [29]. Collectively, these findings highlight coumarin–benzimidazole hybrids as multifunctional anticancer candidates and starting points for developing novel therapeutics against prostate cancer.

### 4.4. 3-(4-Nitrophenyl)coumarin Derivatives as Emerging Anticancer Agents in Prostate Cancer Therapy

**Figure 6:**
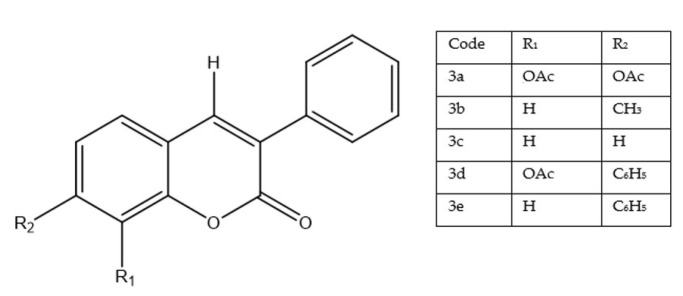
Chemical structure of compounds series 3: **3a–3e**: 3-(4-nitrophenyl)coumarin derivatives with varied R_1_/R_2_ substituents.

A recent study evaluated a panel of 3-(4-nitrophenyl)coumarin derivatives against human prostate cancer PC-3 cells using a crystal violet assay after 48 h exposure (Figure 6). Among the tested compounds, only **3a**—bearing acetoxy substituents at C-7 and C-8—displayed meaningful activity (CC_50_ = 18.2 µM), whereas the remaining analogues (3b–3e) were weak or inactive (CC_50_ > 100 µM) [30].

Mechanism of action effect linked to disruption of cell-cycle progression [30]. In parallel, treatment with 3a caused a dose-dependent decline in mitochondrial membrane potential (ΔΨm), implicating activation of the intrinsic (mitochondrial) apoptotic pathway. Compared with reference treatments, 3a was less potent than docetaxel—a first-line chemotherapeutic in prostate cancer—but substantially more active than unmodified coumarin, which showed no cytotoxic effect in this model [31]. Notably, cytotoxicity toward normal cells was observed, raising concerns about selectivity and safety that warrant targeted optimization. The activity pattern underscores how specific substitutions on the coumarin scaffold—here, 7,8-diacetoxy—can dramatically alter biological responses. Although 3a does not yet rival established agents in efficacy or safety, its distinct profile positions it as a lead structure for systematic optimization (e.g., fine-tuning of ester/ether groups, polarity, and metabolic stability) toward improved potency–selectivity balance in prostate cancer therapy [32].

### 4.5. Linear Furanocoumarin Hybrids as Emerging Anticancer Agents in Breast and Prostate Cancer Therapy

**Figure 7:**
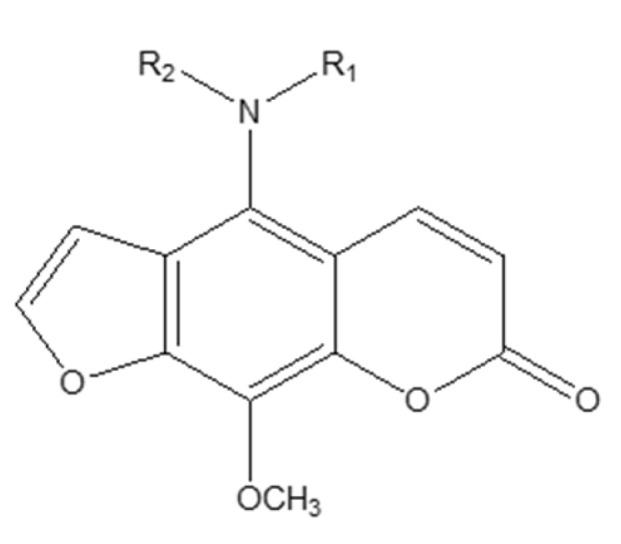
Chemical structure of compounds series 4: **4a**: R_1_ = R_2_ = benzyl; **4b**: R_1_ = R_2_ = CH_2_CH=C(CH_3_)_2_.

To expand therapeutic options for prostate cancer, a series of linear furanocoumarin derivatives was designed by modifying the natural imperatorin scaffold, with substitutions introduced primarily at C4 and C9 (alkyl, aryl, and heteroaryl groups) to probe their impact on anticancer activity (Figure 7). In PC-3 cells, several analogues showed notable antiproliferative effects; in particular, compound **4a** and compound **4b** achieved IC_50_ values of 1.0 µM and 5.0 µM, respectively—surpassing both the parent imperatorin and the reference tamoxifen citrate (IC_50_ = 1.6 µM) used in the study [33].

N-alkylation at C4 emerged as a key driver of activity, whereas O-alkyl or O-acyl modifications generally yielded minimal effects, underscoring the importance of nitrogen substitution at this position. The superior efficacy of the most active analogues is consistent with bulky, hydrophobic moieties that may improve cellular uptake and stabilize interactions with biological targets. Although certain derivatives also exhibited cytotoxicity toward non-malignant cells—highlighting a need for enhanced selectivity—the overall SAR supports C4 N-alkylated furanocoumarins as promising lead structures for further, target-oriented optimization in prostate cancer therapy [34].

### 4.6. Antiproliferative Activity of 8-Isopentenyloxy Coumarin in Prostate Cancer Cells

The cytotoxic potential of 8-isopentenyloxy coumarin was investigated in PC-3 prostate cancer cells across three time points (24, 48, and 72 h), with cell viability measured using the Alamar Blue assay. The IC_50_ values were 29.73 µg/mL at 24 h, increased to 51.14 µg/mL at 48 h, and then decreased to 24.57 µg/mL at 72 h, indicating a time-dependent profile in which prolonged exposure ultimately enhanced cytotoxicity. The transient rise in IC_50_ at 48 h may reflect short-term cellular adaptation or partial tolerance, followed by restoration of sensitivity upon extended treatment. Overall, the marked reduction in IC_50_ at 72 h underscores the importance of exposure duration for maximizing antiproliferative effects. Together with the need to clarify the mechanism and improve selectivity, these observations position 8-isopentenyloxy coumarin as a lead candidate that merits further mechanistic and in vivo evaluation in prostate cancer models [35].

### 4.7. Evaluation of Coumarin-Based Compounds in PC-3 Prostate Cancer Cells

**Figure 8:**
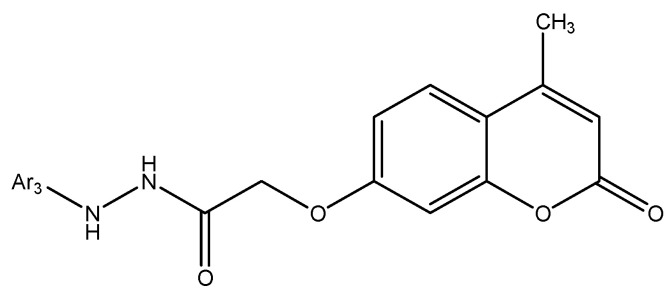
Chemical structure of compound **5**.

A library of twenty-five newly synthesized coumarin derivatives was profiled against PC-3 prostate cancer cells. Although these scaffolds previously showed antiproliferative effects in MCF-7 breast cancer models, they did not translate into comparable activity in PC-3, indicating that the current chemotypes may not effectively engage survival pathways relevant to prostate cancer biology [36]. Within the set, compound **5** (Figure 8) was notable for potent VEGFR2 kinase inhibition and compliance with multiple drug-likeness criteria; nevertheless, it failed to elicit measurable cytotoxicity in PC-3 cells, suggesting that VEGFR2 is unlikely to be a dominant survival driver in this line [11].

**Figure 9:**
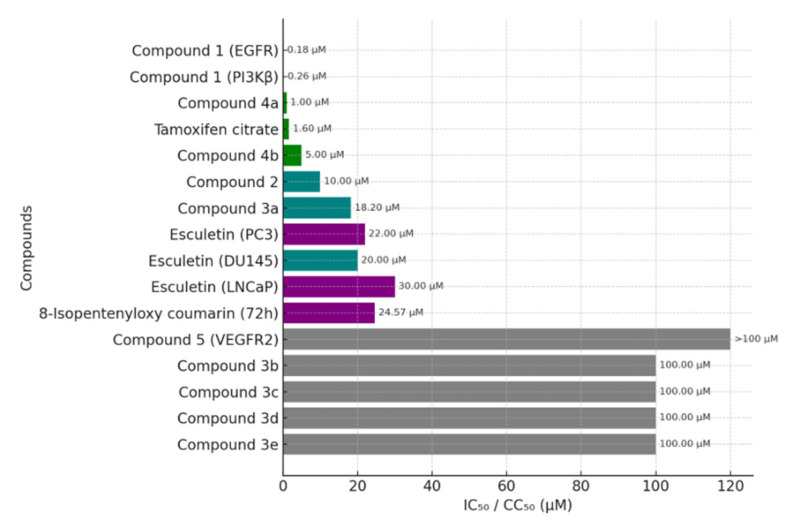
Comparative IC_50_/CC_50_ values of coumarin derivatives in prostate cancer models.

Overall, the data underscore cancer-type specificity in discovery campaigns and argue for prostate-cancer-tailored strategies—either via structure optimization of coumarin cores or redirection toward alternative, disease-relevant targets—guided by deeper molecular profiling of PC-3 cells [37]. As summarized in Figure 9 potency varied widely across exemplars from this review: compound **1** displayed submicromolar kinase inhibition (EGFR 0.18 µM; PI3Kβ 0.26 µM) and outperformed tamoxifen citrate (1.6 µM), compounds **4a** (1.0 µM) and **4b** (5.0 µM) were also strong performers, compound **2** (~10 µM) and compound **3a** (18.2 µM) showed moderate activity, natural derivatives such as esculetin (≈20–30 µM) and 8-isopentenyloxy coumarin (24.57 µM) were comparatively less active, whereas compound **5** and compounds **3b–3e** were essentially inactive in PC-3 (>100 µM).

## 5. Coumarin-Based Derivatives for Targeted Lung Cancer Therapy

Within lung cancer—predominantly NSCLC (e.g., A549, H1299, H460, H2170)—coumarin scaffolds have been investigated as modulators of EMT/motility, topoisomerase I and CDK2 activity, Hsp90 chaperone function, and the PI3K/Akt/mTOR pathway, with coordinated effects on apoptosis and autophagy. The subsections below highlight representative chemotypes and link substitution patterns to these pathway-level outcomes. For a concise map of motifs and targets pertinent to this section, see Section 9, Table 1.

### 5.1. Coumarin Hybrids as Modulators of Epithelial–Mesenchymal Transition and Cell Migration in Lung Cancer Models

**Figure 10:**
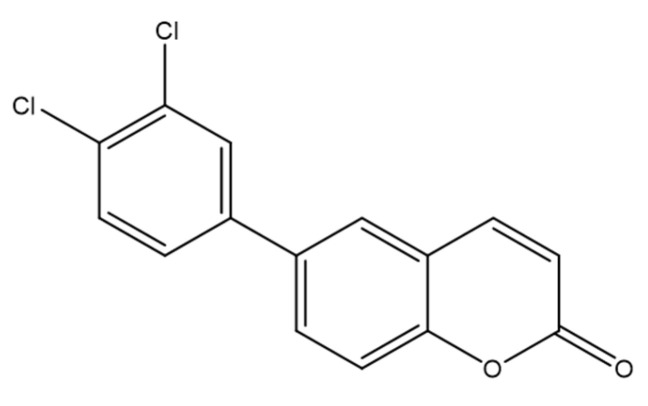
Chemical structure of compound **6**.

To identify new strategies for non-small-cell lung cancer (NSCLC), a series of coumarin-based derivatives and structural analogues was synthesized via palladium-catalyzed cross-coupling from triflate intermediates with phenylboronic acids, terminal alkynes, or organozinc reagents, affording a diversified set of scaffolds. The compounds were profiled for cytotoxicity against A549 and H2170 NSCLC cell lines alongside non-cancerous NIH-3T3 fibroblasts, with cisplatin as a reference [38]. Among them, compound **6** (Figure 10), bearing a 3,4-dichlorophenyl substituent—formally 6-(3,4-dichlorophenyl)-2H-chromen-2-one—showed notable activity, with CC_50_ = 7.1 µM in A549 and 3.3 µM in H2170, while exhibiting a substantially higher CC_50_ in NIH-3T3, consistent with selectivity toward cancer cells.

Mechanistic assays indicated interference with epithelial–mesenchymal transition (EMT): in IL-1β–stimulated A549 cells, treatment induced morphological changes, downregulated vimentin, and disrupted F-actin organization, collectively supporting EMT inhibition [39]. Compound **6** also reduced cell migration in wound-healing assays, aligning with an anti-metastatic profile. Together, these data position compound **6** as a dual-acting candidate that couples cytotoxic potency with EMT/motility modulation, highlighting the promise of coumarin hybrids for further optimization in lung cancer therapy [40].

### 5.2. Coumarin Hybrids as Dual-Acting Topoisomerase Inhibitors with Antiproliferative Potential in A549 Lung Cancer Cells

**Figure 11:**
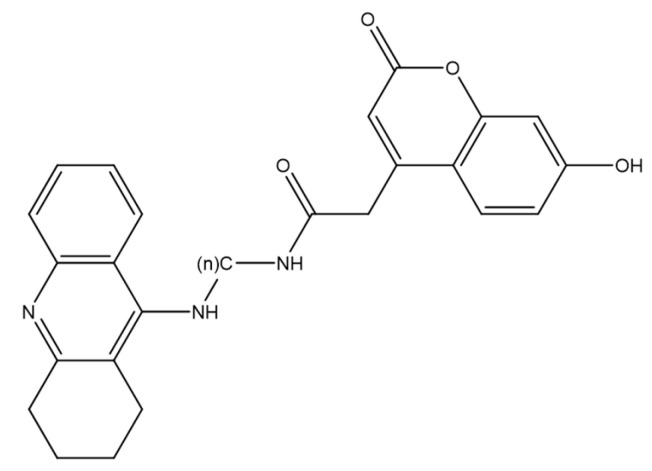
Chemical structure of compounds series 7: **7a**: n = 8; **7b**: n = 9.

A series of tacrine–coumarin hybrids was evaluated as candidate topoisomerase I inhibitors with antiproliferative activity in A549 human lung carcinoma cells (Figure 11). Activity depended strongly on the linker length connecting the tacrine and coumarin motifs: analogues with extended methylene chains, notably 7a and 7b, were the most potent, with IC_50_ = 27.04 µM and 21.22 µM at 48 h, respectively. Across the series, MTT and clonogenic readouts showed a time- and concentration-dependent decrease in viable cell number/metabolic activity, consistent with a cytostatic profile [41].

Flow cytometry indicated G0/G1 arrest, supporting the interpretation that the reduction in cell counts primarily reflects inhibited proliferation rather than frank cytotoxicity [40]. Enzymology demonstrated effective inhibition of human topoisomerase I at 60 µM with no detectable inhibition of topoisomerase IIα, indicating target selectivity [9]. Fluorescence imaging confirmed cellular uptake (diffuse cytoplasmic distribution) for a representative analogue—7d (denoted 1d in the original report)—corroborating intracellular target engagement [42]. Taken together, these findings highlight tacrine–coumarin hybrids, particularly 7c/7d, as promising leads for NSCLC drug discovery, with a mechanism involving topoisomerase I inhibition coupled to cell-cycle modulation [9,41,42].

### 5.3. Novel Coumarin–Piperazine-2(5H)-Furanone Hybrids as Potential Anti-Lung Cancer Agents: Synthesis, Biological Evaluation, and Molecular Docking Studies

**Figure 12:**
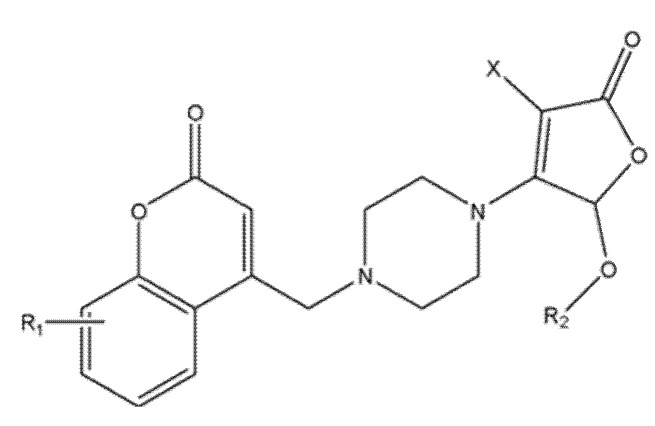
Chemical structure of compound **8** (R_1_ = OH; R_2_ = benzyl; X = Cl).

A novel series of coumarin–piperazine–2(5H)-furanone hybrids was obtained by installing a furanone moiety onto the coumarin scaffold via a piperazine linker and evaluated against A549 lung cancer cells and normal lung fibroblasts (WI-38), with cytarabine (CAR) as the reference [43]. Compound **8** (Figure 12) was the most active (A549 IC_50_ = 11.28 μM), ~18-fold more potent than CAR in this setting, while showing limited toxicity toward WI-38 (IC_50_ = 411.93 μM), yielding a selectivity index (SI) ≈ of 37, markedly exceeding that of the standard drug.

Structure–activity analysis indicated that a bornyl substituent (observed in other synthesized coumarin–piperazine–2(5H)-furanone derivatives) enhances both potency and selectivity [44]. Beyond A549, compound **8** maintained a favorable profile across multiple NSCLC lines (Calu-1, PC-9, H460; IC_50_ 5.72–45.46 μM), with potent activity in H460 (IC_50_ = 5.72 μM; SI ≈ 72). Flow cytometry supported concentration-dependent apoptosis, and docking suggested CDK2 as a plausible target (reported PDB ID: 1HCK), consistent with the observed cell-cycle effects [45]. These data position this analog as a selective, cytotoxic lead candidate that warrants in vivo validation and medicinal-chemistry optimization.

### 5.4. Benzyl Coumarin–Furoxan Derivatives as Inducers of Apoptosis and Autophagy in Non-Small-Cell Lung Cancer

**Figure 13:**
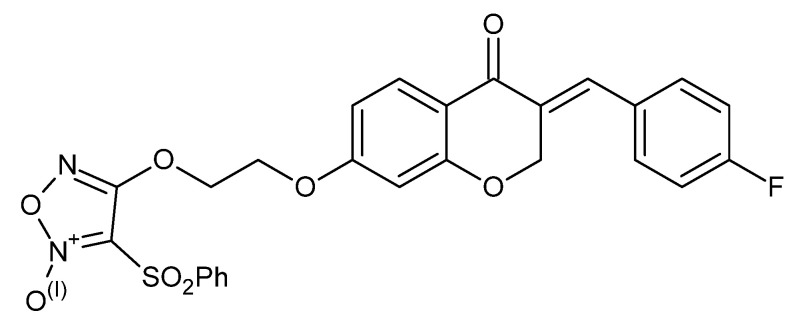
Chemical structure of compound **9**.

A benzyl coumarin seco-B-ring derivative conjugated to a nitric-oxide–releasing phenylsulfonyl furoxan compound **9** (E-3-(4-fluorobenzylidene)-7-(2-((5-hydroxy-4-phenylsulfonyl)-4,5-dihydro-1,2,5-oxadiazol-3-yl)oxy)ethoxy)chroman-4-one) was developed to combine proliferation blockade with modulation of survival signaling in NSCLC. In A549 cells, compound **9** (Figure 13) showed significant growth-inhibitory activity in the MTT assay [46].

Mechanistic studies indicated induction of programmed cell death: flow cytometry confirmed caspase-dependent apoptosis, and complementary approaches (electron microscopy, confocal imaging, Western blotting) demonstrated autophagy activation [47]. Notably, chemical inhibition of autophagy or ATG5 silencing did not appreciably alter cytotoxicity, consistent with nonprotective autophagy in this setting, while apoptosis appears to be the dominant cell death effector. Further analysis implicated the PI3K/Akt/mTOR axis as a critical target; co-treatment with pathway inhibitors (e.g., 3-MA, LY294002) yielded synergistic enhancement of anticancer effects, underscoring pathway convergence and combination potential [48]. Overall, the ability of compound **9** to trigger apoptosis, elicit nonprotective autophagy, and interfere with pro-survival signaling highlights a multitarget profile well suited for NSCLC combination strategies aimed at overcoming resistance.

### 5.5. Coumarin–Matrine Hybrids as Selective Hsp90 (NTD and CTD) Isoform Inhibitors for Lung Carcinoma Therapy

**Figure 14:**
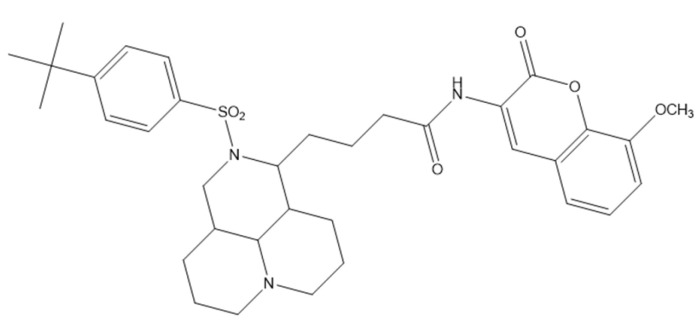
Chemical structure of compound **10**.

A series of matrine–coumarin hybrids was strategically designed as dual inhibitors of the N-terminal (NTD) and C-terminal (CTD) domains of Hsp90, a molecular chaperone frequently overexpressed in lung cancer. The compounds were screened in vitro against A549 (non-small-cell lung cancer), HepG-2 (hepatocellular carcinoma), and HeLa (cervical carcinoma) cells. Compound **10**—4-(2-((4-(tert-butyl)phenyl)sulfonyl)decahydro-1H,4H-pyrido [3,2,1-ij][1,6]naphthyridin-1-yl)-N-(8-methoxy-2-oxo-2H-chromen-3-yl)butanamide (Figure 14)—emerged as the most promising analogue, showing potent cytotoxicity (IC_50_ 7.35 ± 0.097 µM in A549; 7.72 ± 0.10 µM in HepG-2; 8.02 ± 0.065 µM in HeLa) with negligible toxicity toward BEAS-2B normal lung epithelial cells [49].

Mechanistic assays in A549 cells showed suppressed proliferation, reduced colony formation, and impaired migration, consistent with disruption of Hsp90 chaperone function. In vivo, in A549 xenograft–bearing BALB/c nude mice, compound **10** achieved 72.4% tumor growth inhibition (TGI), outperforming 5-fluorouracil (64.3%) and the parent matrine (46.8%) [50]. Molecular docking supported high-affinity binding to both Hsp90 domains (NTD PDB: 3T0Z; CTD PDB: 2CG9), with stabilizing interactions that included hydrogen bonds (Gly137, Asn51, Glu453), CH–π and π–anion contacts, and van der Waals forces [51]. These data position compound **10** as a dual-domain Hsp90 inhibitor with compelling preclinical efficacy and selectivity, meriting further optimization and in vivo development as a targeted agent for lung cancer.

### 5.6. Coumarin–Phenylsulfonyl Furoxan Hybrids as Modulators of Programmed Cell Death Through Apoptosis and Autophagy in Lung Adenocarcinoma (A549)

**Figure 15:**
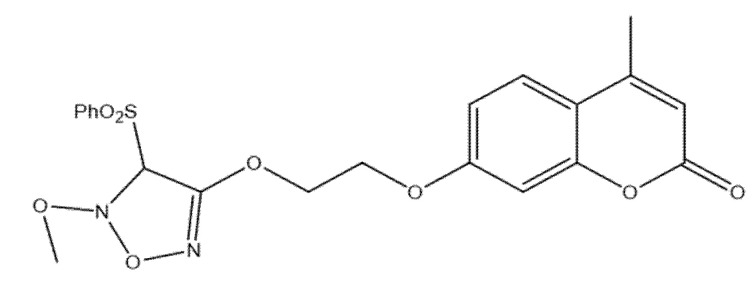
Chemical structure of compound **11**.

Among newly developed coumarin–phenylsulfonyl furoxan hybrids, compound **11**—7-(2-((5-methoxy-4-(phenylsulfonyl)-4,5-dihydro-1,2,5-oxadiazol-3-yl)oxy)ethoxy)-4-methyl-2H-chromen-2-one (Figure 15)—exhibited notable antiproliferative effects against A549 and H1299 lung adenocarcinoma cells.

Mechanistic profiling indicated that the compound regulates cancer-cell fate via dual, intersecting programs: it induces apoptosis, evidenced by caspase-3 activation and PARP cleavage together with downregulation of BCL-2, while concurrently triggering autophagy, as shown by autophagosome accumulation, elevated LC3-II, and increased autophagic flux [17]. Blocking autophagy using 3-MA, chloroquine, or ATG5 siRNA further enhanced cell death, indicating that the autophagy elicited by compound **11** is protective and counterbalances its pro-apoptotic activity. At the signaling level, the compound downregulated the Akt/mTOR axis—an established negative regulator of autophagy—and activated ERK1/2, a promoter of autophagic responses [52]. Together, these effects position compound **11** as a potent coumarin–furoxan hybrid that drives apoptosis while eliciting protective autophagy; significantly, its efficacy may be augmented in combination with autophagy inhibitors, offering a rational strategy to overcome resistance mechanisms in lung cancer therapy [52].

### 5.7. Coumarin–Pyrazole Carbodithioate Hybrids as Potent CDK2 Inhibitors for Lung Carcinoma Therapy (A549; PDB: 1DI8)

**Figure 16:**
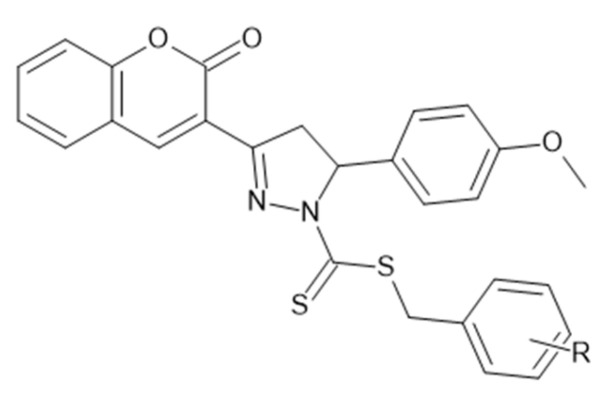
Chemical structure of compound **12**: (R = Br).

Coumarin–pyrazole–linked carbodithioate derivatives were profiled against A549 lung cancer cells by MTT with cisplatin as a benchmark. A clear halogen-driven SAR emerged: the para-bromo analogue (Figure 16) was most potent (IC_50_ = 2.88 µM), followed by the meta-bromo (3.12 µM) and para-chloro (6.05 µM) congeners; in contrast, methyl/methoxy variants showed only moderate cytotoxicity, and nitro analogues were markedly less active [53]. To rationalize these trends, CDK2 was examined as a putative target: docking to the ATP site of CDK2 (PDB: 1DI8) supported strong binding for the most active halogenated analogues and stable interactions consistent with their biological rank order, whereas weaker binders mapped to the less active series [53].

Beyond activity, selected members displayed favorable physicochemical robustness, with measured pK_a_ ≈ 5.6–6.8 and photostability across pH 3.3–9.2, features compatible with downstream formulation work [54]. Together, these data position coumarin–pyrazole carbodithioates as versatile, chemically stable leads for NSCLC discovery campaigns, combining CDK2 engagement with tunable potency and properties amenable to optimization [53,54].

### 5.8. Azaheterocyclic Coumarin Derivatives as Multitargeted Kinase Inhibitors Induce Apoptosis in Non-Small-Cell Lung Carcinoma (A549; PDB: 2OH4)

**Figure 17:**
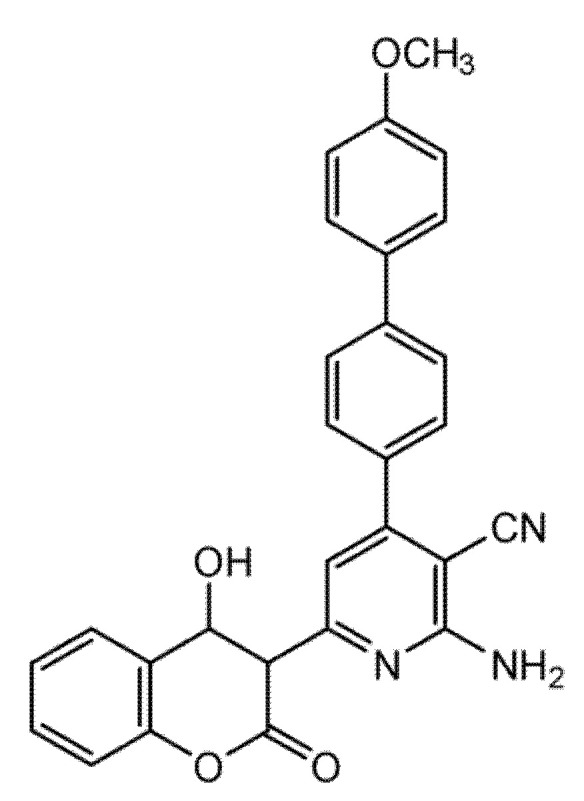
Chemical structure of compound **13**.

Recent work on azaheterocyclic coumarin derivatives identified multiple candidates with activity against A549 non-small-cell lung cancer (NSCLC) cells, with ten of the twenty synthesized molecules surpassing the reference staurosporine (IC_50_ = 9.50 µM) in potency. Compound **13** (Figure 17) emerged as the lead (IC_50_ = 1.05 µM), and selectivity assays in normal lung fibroblasts (WI-38) indicated markedly attenuated toxicity for the related analogue 13b (IC_50_ = 53.76 µM), together supporting a favorable therapeutic window [17]. Mechanistic profiling in A549 revealed a pro-apoptotic phenotype: compound **13** provoked cell-cycle perturbations (increases in pre-G1 and S-phase populations) and robust apoptosis, including a ~24-fold rise in pre-G1 content and 22.37% late apoptotic cells by flow cytometry [52].

Beyond cytotoxic and apoptotic effects, the series displayed broad kinase inhibition at submicromolar levels across angiogenesis-linked targets—VEGFR-2, PDGFR, FGFR, and EGFR—consistent with a multitarget mode of action; molecular docking supported strong binding of 13b to VEGFR-2 (PDB: 2OH4; docking score –7.0594 kcal/mol) [55]. An in silico ADME appraisal (SwissADME) further suggested drug-like behavior for 13b—high gastrointestinal absorption, Lipinski compliance, and no PAINS alerts—pointing to oral developability with minimal assay interference [55]. The potency, kinase-inhibition spectrum, induction of apoptosis, and predicted pharmacokinetic features position compound **13** as a compelling multitarget NSCLC lead meriting further preclinical optimization and validation [17,52,55].

### 5.9. Coumarin-Based 1,2,3-Triazole-Linked Hybrids as Promising Anticancer Agents Against Lung Cancer: Design, Synthesis, and Cytotoxicity Evaluation

**Figure 18:**
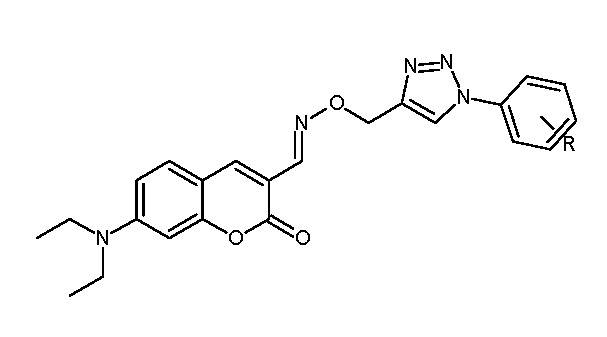
Chemical structure of compounds **14a** (R = p-Br) and **14b** (R = o-OMe).

In pursuit of selective non-small-cell lung cancer (NSCLC) therapies, a series of coumarin–1,2,3-triazole hybrids was synthesized via a multistep route (Vilsmeier–Haack formylation → oxime formation → copper-catalyzed azide–alkyne cycloaddition) and investigated for interactions with MST3 (PDB: 4QMP) by molecular docking. The protocol was validated by redocking the co-crystallized ligand DKI (RMSD 1.025 Å), supporting model reliability. Among the new derivatives, 14a (para-bromo) and 14b (ortho-methoxy) achieved the most favorable docking scores, stabilized by hydrogen bonds and π–π stacking within the kinase site [56] (Figure 18).

**Figure 19:**
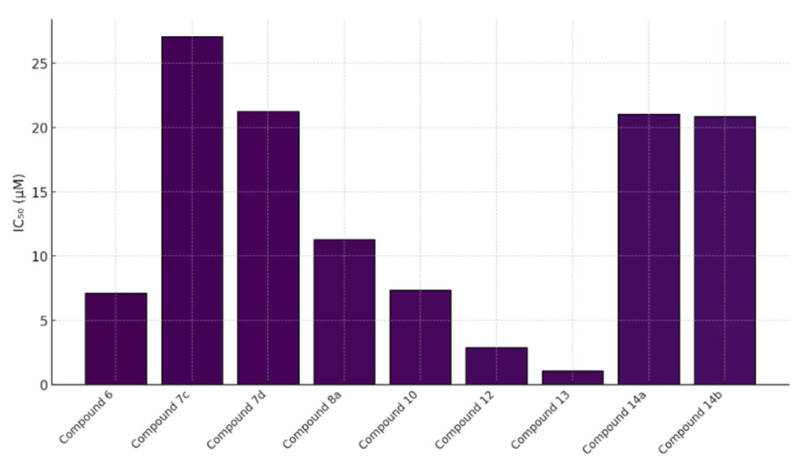
Comparison of IC_50_ values for coumarin-based derivatives tested against A549 lung-cancer cells.

In MTT assays against A549 cells, 14a showed IC_50_ = 21.03 ± 1.22 μM; 14b exhibited potency comparable to doxorubicin (IC_50_ = 20.82 ± 1.16 μM) while maintaining low toxicity toward HEK-293 cells, indicating a favorable selectivity profile. Substituent electronics and placement modulated MST3 engagement—e.g., the p-bromo group in 14a and the o-methoxy group in 14b promoted productive contacts with electron-rich residues in the active site [57]. A cross-compound comparison of A549 IC_50_ values further contextualizes these results (Figure 19): compound **13** (1.05 μM) and compound **12** (2.88 μM) were most active; compounds **6** (7.1 μM), 10 (7.35 μM), and **8a** (11.28 μM) showed moderate activity; whereas 7c, 7d, 14a, and 14b fell in the ~20–27 μM range—together supporting 12 and 13 as leading candidates for further development.

## 6. Coumarin-Based Derivatives for Targeted Breast Cancer Therapy

Within breast cancer—spanning ER-positive (e.g., MCF-7, T47D) and triple-negative models (e.g., MDA-MB-231, MDA-MB-468)—coumarin scaffolds have been evaluated as modulators of aromatase/ER and EGFR signaling, VEGFR-2/CCL2-linked angiogenesis, and the PI3K/Akt/mTOR pathway, with reproducible caspase-mediated apoptosis and G1/G2–M arrest; context-dependent autophagy and MDR/P-gp effects are also noted. The subsections below summarize representative chemotypes and connect substitution patterns to these pathway-level phenotypes. For a concise map of motifs and targets pertinent to this section, see Section 9, Table 1.

### 6.1. Coumarin Hybrids as Promising VEGFR-2 Inhibitors: Molecular Modeling and Anticancer Potential Against Breast Cancer

**Figure 20:**
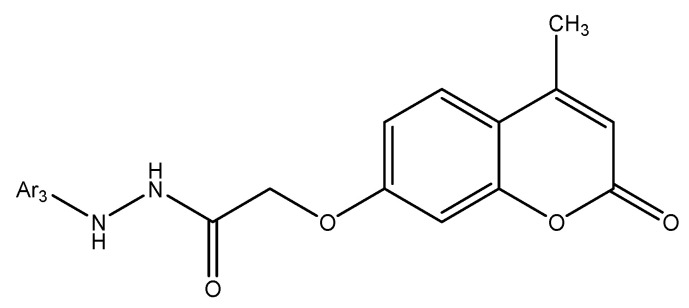
Chemical structure of compound **15**.

A library of twenty-five newly synthesized coumarin derivatives showed notable anticancer activity in MCF-7 breast cancer cells, with several members outperforming the reference staurosporine; compound **15** (Figure 20) was the most potent (IC_50_ = 1.24 µM vs. 1.65 µM for staurosporine), nominating it as a lead for further study [36].

Mechanistic interrogation implicated VEGFR-2 inhibition as a driver of efficacy; within the set, compound **14** displayed potent biochemical VEGFR-2 inhibition (IC_50_ = 0.36 µM), while cellular analyses for compound **15** demonstrated G2/M arrest, caspase-9 activation, and a marked increase in the pre-G1 population, consistent with apoptosis induction [58]. Molecular docking further supported VEGFR-2 engagement by compound **15**, revealing stable interactions within the kinase active site (PDB: 4ASD) and a geometry compatible with the observed activity [59]. In silico ADME profiling highlighted favorable, drug-like properties for compound **14**—including high predicted gastrointestinal absorption, Lipinski compliance, and absence of PAINS alerts—suggesting oral developability with low assay interference [60]. Collectively, these results position compound **15** as a VEGFR–2–targeted breast cancer lead that combines potent cytotoxicity with apoptosis induction and angiogenesis blockade, while compound **14** emerges as a complementary VEGFR-2-focused chemotype with encouraging pharmacokinetic prospects warranting in vivo exploration [36,58,59,60].

### 6.2. Coumarin Hybrids as Promising Dual-Target Agents Against Breast and Cervical Cancer Through Modulation of VEGFR-2 and p38α MAPK Pathways

**Figure 21:**
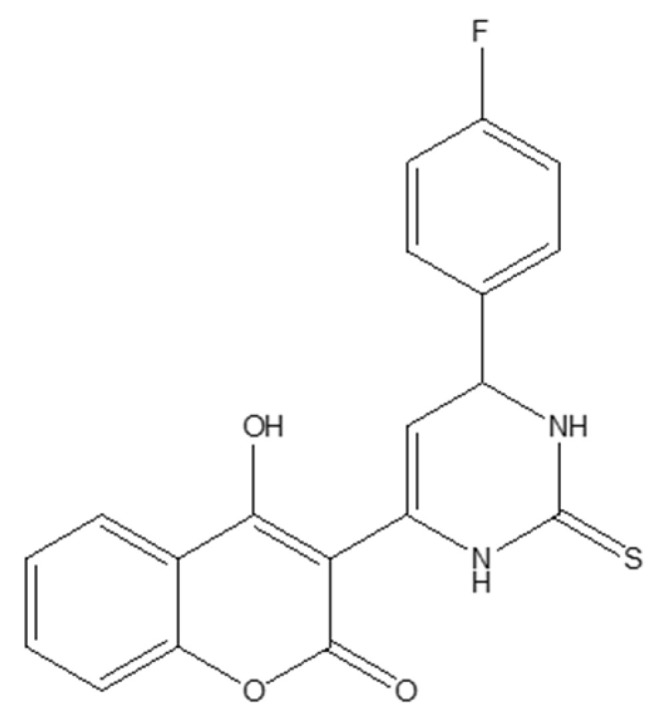
Chemical structure of compound **16**.

Fluorinated coumarin scaffolds were recently engineered to engage multiple oncogenic pathways relevant to breast and cervical cancers. Strategic substitutions at the 3- and 4-positions of the coumarin nucleus with bioactive groups enhanced biological performance, yielding derivatives with improved antiproliferative activity and kinase modulation [61]. Among these, compound **16** (Figure 21) emerged as the most active, displaying potent effects against MCF-7 cells (IC_50_ = 7.90 µg/mL) together with potent VEGFR-2 inhibition (94%).

Molecular docking against VEGFR-2 (PDB: 3U6J) supported target engagement, revealing a stable pose within the ATP-binding cleft featuring hydrogen bonds, π–π stacking, and arene–cation contacts with key residues that rationalize the biochemical readouts [62]. Beyond angiogenesis signaling, compound **16** also inhibited p38α MAPK, a kinase implicated in proliferation, migration, and survival, underscoring a dual-target profile with potential to counter pathway redundancy. A complementary QSAR analysis yielded a predictive model (R^2^ = 0.76969; RMSE = 0.10446) consistent with the experimentally observed structure–activity relationships, further supporting the design rationale [63]. Collectively, the potency in MCF-7, robust VEGFR-2 and p38α MAPK modulation, and coherent computational validation position compound **16** as a promising fluorinated coumarin hybrid for multitarget therapy in breast and cervical cancers, warranting in vivo evaluation and pharmacokinetic studies to assess translational potential.

### 6.3. 4,7-Disubstituted Coumarin Derivatives as Dual Aromatase and EGFR Inhibitors in Breast Carcinoma (MCF-7, MDA-MB-231; PDB: 3EQM, 1M17)

**Figure 22:**
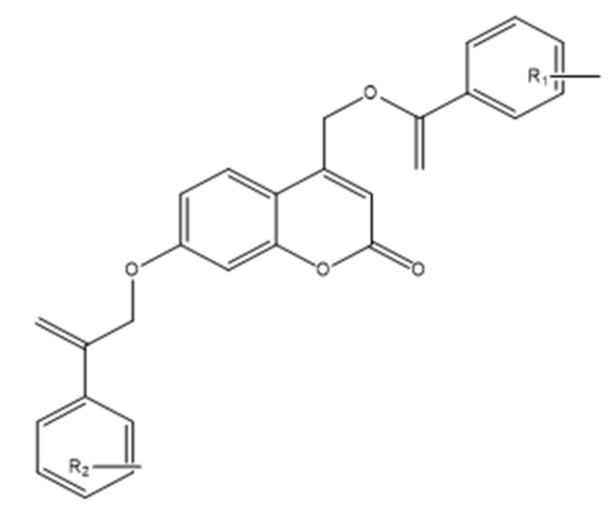
Chemical structures of compounds 17 series: **17a**: R_1_ = R_2_ = 4-H; **17b**: R_1_ = 4-H, R_2_ = Cl; **17c**: R_1_ = 4-Cl, R_2_ = H; **17d**: R_1_ = R_2_ = 4-Cl; **17e**: R_1_ = 2-OCH_3_, R_2_ = H; **17f**: R_1_ = 2-OCH_3_, R_2_ = Cl.

A series of 4,7-disubstituted coumarins bearing a methyl ester at C4 was designed to probe therapeutic relevance across MCF-7 (ER-positive) and MDA-MB-231 (triple-negative) breast cancer models, with MCF-10A normal epithelial cells as a comparator and doxorubicin as the reference (Figure 22). In MTT assays, several analogues showed marked activity in MCF-7; notably, two compounds achieved IC_50_ values comparable to doxorubicin, nominating this chemotype for deeper mechanistic inquiry.

Follow-up studies indicated that 17b, 17d, and 17f induced cell-cycle arrest (G0/G1 and S) and apoptosis, evidenced by elevated caspase-9 and a favorable BAX/BCL-2 ratio [64]. Biochemical profiling revealed dual target engagement within the series: 17b acted as a potent aromatase inhibitor, reaching ~95% of exemestane activity, whereas 17d and 17f showed potent EGFR inhibition approaching that of erlotinib, together supporting efficacy across receptor-positive and triple-negative contexts [65]. Molecular docking corroborated these findings, showing stable binding in the active sites of aromatase (PDB: 3EQM) and EGFR (PDB: 1M17), while complementary ADMET predictions and QSAR analysis supported favorable pharmacokinetics and suggested that judicious tuning of hydrophilic–hydrophobic substitutions could further enhance activity and selectivity [58]. Overall, the data position 4,7-disubstituted coumarins as a multitarget breast cancer lead, warranting in vivo validation and toxicity assessment.

### 6.4. Neo-Tanshinlactone–Chalcone Coumarin Hybrids as TNF-α–Targeted Anticancer Agents in Breast Carcinoma (MCF-7, MDA-MB-231; PDB: 2AZ5)

**Figure 23:**
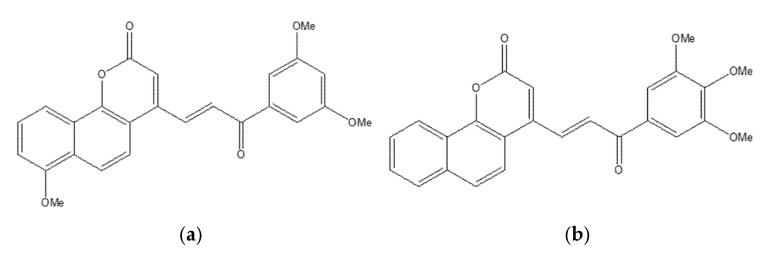
Chemical structure of compounds (**a**) **18a** and (**b**) 1**8b**.

A novel series of neo-tanshinlactone–chalcone hybrids was synthesized via Horner–Wadsworth–Emmons (HWE) olefination from 4-formyl-2H-benzo[h]chromen-2-one and the corresponding phosphonate, then evaluated by MTT across four human cancer cell lines—MCF-7 (ER-positive), MDA-MB-231 (ER-negative), HeLa (cervical), and Ishikawa (endometrial)—with tamoxifen as the reference. The series showed moderate-to-high cytotoxicity (IC_50_ ≈ 6.8–19.2 µM); compound **18a** was most potent (IC_50_ = 6.8 µM in MCF-7; 8.5 µM in MDA-MB-231), outperforming tamoxifen in this panel, while **18b** displayed selective activity against breast cancer lines (14.4 µM in MCF-7; 15.7 µM in MDA-MB-231) (Figure 23) [66]. Notably, none of the hybrids were cytotoxic to HEK-293 normal cells, indicating a favorable therapeutic index.

Molecular docking implicated tumor necrosis factor-α (TNF-α; PDB: 2AZ5) as a putative target: 18b formed a stable complex featuring hydrogen bonds and complementary noncovalent contacts within the cytokine interface, consistent with the proposed mechanism wherein TNF-α signaling can promote breast-cancer progression (e.g., via aromatase upregulation). ADMET profiling supported developability for 18a/18b, including Lipinski compliance, high predicted oral bioavailability, acceptable blood–brain barrier permeability, and generally favorable pharmacokinetics. These findings nominate 18a as a TNF-α–targeted lead for breast cancer therapy, with 18b providing additional SAR leverage for the next generation of coumarin–chalcone hybrids [67].

### 6.5. Fluorinated Coumarin Derivatives as Dual VEGFR-2 and p38α MAPK Inhibitors in Breast and Cervical Carcinoma (MCF-7, HeLa; PDB: 3U6J, 3FMK)

**Figure 24:**
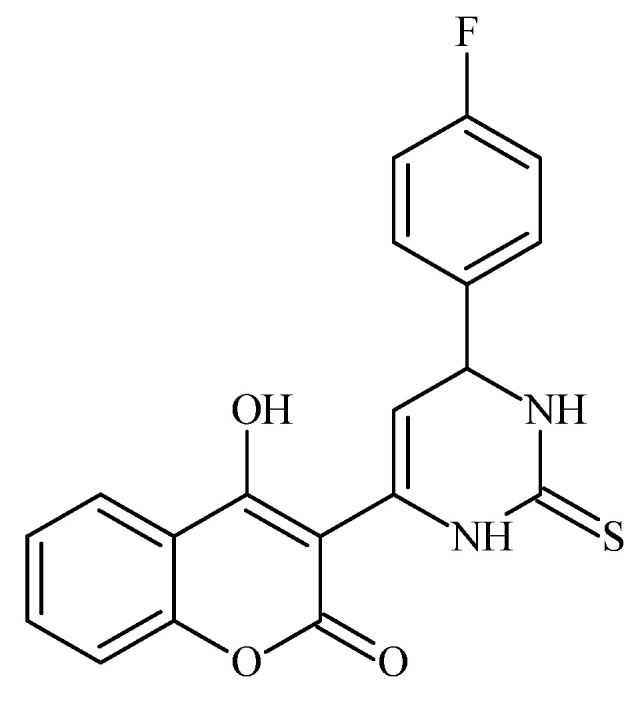
Chemical structure of compound **19**.

Fluorinated coumarin scaffolds were synthesized with diverse bioactive substituents at the 3- and/or 4-positions and profiled for dual kinase modulation—VEGFR-2 and p38α MAPK—alongside antiproliferative activity in MCF-7 (breast) and HeLa (cervical) cells. Most analogues showed moderate inhibition of VEGFR-1/VEGFR-2 and measurable effects in MCF-7, while compound **19** (a thioxopyrimidine-based derivative, Figure 24) displayed a pronounced preference for p38α MAPK (PDB: 3FMK) [61].

In HeLa cells, 19 reduced viability to 80% and 72% at 10 and 30 μM, respectively, with performance comparable to tamoxifen in this assay. Molecular docking supported target engagement, revealing stable poses in VEGFR-2 (PDB: 3U6J) and p38α MAPK, stabilized by hydrogen bonds, π–π stacking, and arene–cation contacts. A complementary QSAR model (R^2^ = 0.76969; RMSE = 0.10446) highlighted physicochemical determinants—molecular weight, logP, and surface-area descriptors—correlating with activity. Taken together, these data nominate fluorinated coumarins, particularly compound **19**, as a promising multitarget chemotype combining VEGFR-2 and p38α MAPK modulation with demonstrable antiproliferative effects, warranting in vivo evaluation and pharmacokinetic optimization for breast and cervical cancer applications [63].

### 6.6. Coumarin–Pyrimidine–Triazole Hybrids as Multitargeted Computational Leads for Breast Carcinoma (MCF-7; PDB: 3SRQ, 4KD7; Tubulin Colchicine/Vinblastine Sites)

**Figure 25:**
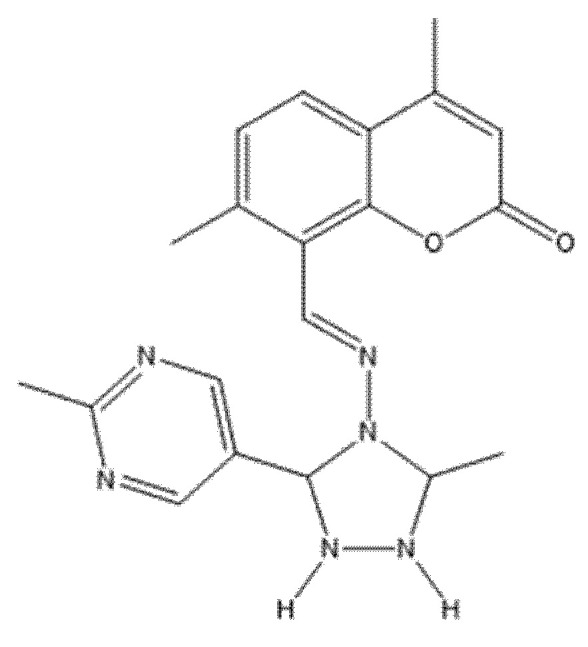
Chemical structure of compound **20**.

Computational profiling of coumarin–pyrimidine–triazole hybrids against MCF-7 breast carcinoma yielded four statistically robust QSAR models (R^2^ > 0.97; Q^2^ > 0.94), with Williams plot diagnostics used to flag and exclude outliers (e.g., compound **20**), improving predictive reliability [68]. Key descriptors (e.g., MAXDP, JGI7, and charge-distribution terms) correlated positively with activity, whereas AATS7s and MATS2c showed negative correlations; guided by these trends, twelve new designs were proposed, most with predicted pIC_50_ > 8.0, surpassing the top compound from the original set (pIC_50_ = 5.809) [69]. Molecular docking indicated stable interactions with Staphylococcus aureus DHFR (PDB: 3SRQ), human DHFR (PDB: 4KD7), and the colchicine- and vinblastine-binding sites on tubulin; several hybrids displayed favorable binding energies (–8.6 to –9.1 kcal/mol), and notably compound **20** (Figure 25)—despite its QSAR outlier status—showed the strongest affinity at the colchicine site (–9.8 kcal/mol) via hydrogen bonds, π–π stacking, van der Waals contacts, and halogen bonding [69].

Complementary in silico pharmacology suggested that these chemotypes could modulate aromatase and ERα (including its ligand-binding domain) with profiles comparable to tamoxifen, while ProTox-II projected favorable safety; SwissADME predicted good oral bioavailability (scores > 0.55), moderate-to-high solubility (log S −6 to −2), reasonable synthetic accessibility (3.16–3.71), absence of CNS penetration, and limited CYP liabilities largely confined to CYP1A2 and CYP3A4 [70]. Taken together, these data nominate the coumarin–pyrimidine–triazole scaffold as a multitarget computational lead for breast-cancer therapy, with convergent QSAR/docking/ADME evidence that justifies experimental validation and iterative medicinal-chemistry optimization [68,69,70].

### 6.7. Coumarin-Based Derivatives as Caspase-9 and BCL-2 Modulators Inducing Apoptosis in Breast Carcinoma (MCF-7; PDB: 1NW9, 4MAN)

**Figure 26:**
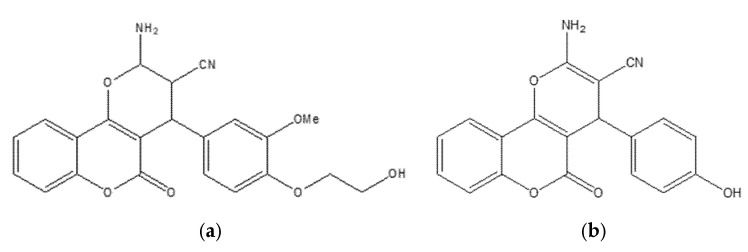
Chemical structures of compounds (**a**) **21a** and (**b**) **21b**.

Two newly synthesized coumarin-based derivatives, **21a** and **21b** (Figure 26), were evaluated in vitro in MCF-7 cells and in vivo in BALB/c mice bearing breast-tumor implants. Both compounds showed notable potency (IC_50_ = 9.5 µM for 21a; 12.3 µM for 21b), comparable to doxorubicin in this experimental context.

Gene-expression analyses (qRT-PCR) supported activation of the intrinsic (mitochondrial) apoptotic pathway, with upregulation of caspase-9 alongside downregulation of BCL-2, c-Myc, and COX-2 [71]. Histopathology of tumor, liver, and lung from treated animals indicated reduced tumor aggressiveness and metastatic spread, with the hydroxyethoxy–methoxy–substituted analogue exhibiting the most pronounced effects. Molecular docking was concordant with these findings, revealing strong binding of the lead compound to caspase-9 (PDB: 1NW9) and BCL-2 (PDB: 4MAN), consistent with modulation of key apoptotic checkpoints. The selective activity, favorable tolerability, and robust pro-apoptotic profile position these coumarin derivatives as promising candidates for further development in breast cancer chemotherapy; comprehensive preclinical (and ultimately clinical) studies are warranted to establish therapeutic potential and safety [72].

### 6.8. Coumarin–1,3,4-Oxadiazole Hybrids as Estrogen-Receptor Modulators in Breast Carcinoma (MCF-7, MDA-MB-231; PDB: 3ERT)

**Figure 27:**
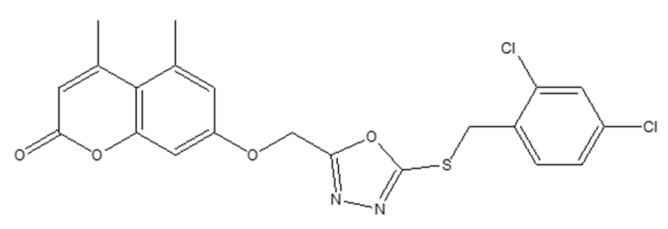
Chemical structures of compound **22**.

Coumarin-linked 1,3,4-oxadiazole hybrids were designed to enhance antiproliferative activity across MCF-7 (ER^+^) and MDA-MB-231 (ER^−^) models. Several analogues were active; 22 (bearing a 2,4-dichlorobenzyl moiety) achieved IC_50_ < 5 µM in MCF-7—~1.4× more potent than tamoxifen in the same assay—while other analogs (with sulfone groups) inhibited MDA-MB-231 at IC_50_ ≈ 7 µM. SAR trends suggested that benzyl substitution boosts potency in ER^+^ settings, whereas sulfone functionality contributes to activity in ER^−^ cells (Figure 27) [73].

**Figure 28:**
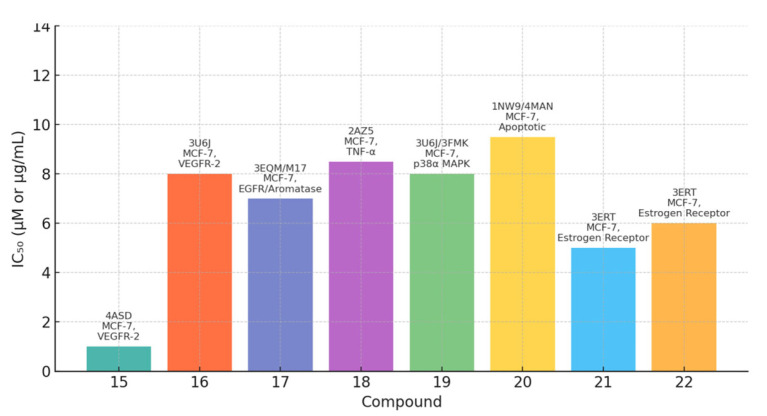
IC_50_ values of coumarin-based derivatives (15–22) against MCF-7 cells with nominated molecular targets (e.g., VEGFR-2, EGFR/aromatase, TNF-α, p38α MAPK, apoptotic signaling, ER).

Docking to ERα (PDB: 3ERT) indicated favorable hydrogen-bonding and hydrophobic contacts consistent with receptor modulation and potential utility where endocrine responsiveness is diminished [74]. A target-oriented summary of activities for the broader 15–22 series in MCF-7 is provided for context (Figure 28).

**Figure 29:**
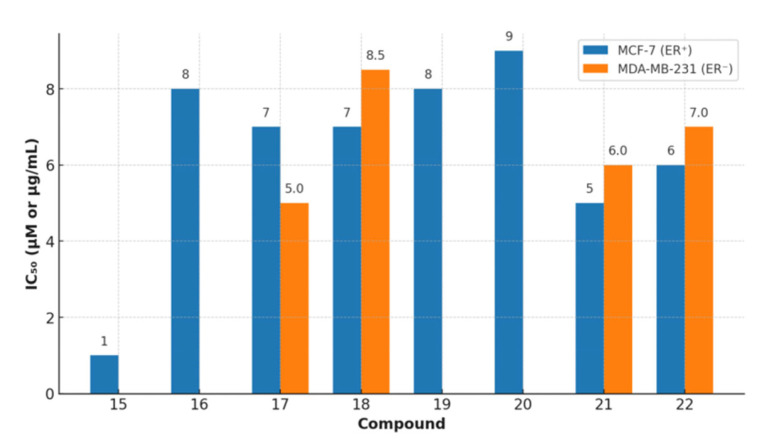
Comparative IC_50_ values of coumarin-based derivatives (15–22) in MCF-7 (ER^+^) and MDA-MB-231 (ER^−^) cell lines.

Bridging these observations to receptor status, the oxadiazole hybrids occupy a niche within the coumarin toolkit that emphasizes ER-proximal engagement in MCF-7. At the same time, selected electronic features (e.g., sulfones) retain residual efficacy in MDA-MB-231 despite the absence of hormone signaling. This positioning complements angiogenesis- and kinase-oriented chemotypes (e.g., VEGFR-2/p38α modulators) and apoptosis-centric designs, suggesting routes for dual-context optimization or rational combinations that pair ER modulation with non-endocrine targets to address pathway redundancy (Figure 29).

Across datasets, triple-negative lines (e.g., MDA-MB-231) tend to be more sensitive to EGFR-biased or mitochondrial-stress coumarins, whereas ER-positive MCF-7 often favors designs with aromatase engagement; this supports subtype-aware prioritization and biomarker-guided selection in future lead optimization [64].

## 7. Coumarin-Based Derivatives for Targeted Blood Cancer Therapy

Within blood cancers—predominantly leukemia (e.g., K562)—coumarin-based designs, particularly benzofuran–chromone–coumarin hybrids, have been explored as tunable modulators of cell-cycle checkpoints (G1, S, G2/M) and caspase-dependent apoptosis, with contributory effects on PI3K/Akt and MAPK signaling. The subsection below illustrates how subtle R-group electronics/sterics redirect phase arrest and amplify apoptotic output, informing potency and selectivity optimization in hematologic settings. For a concise map of motifs and targets pertinent to this section, see Section 9, Table 1.

### Cytotoxic Potential of Benzofuran–Chromone–Coumarin Hybrids Against Leukemia Cells

**Figure 30:**
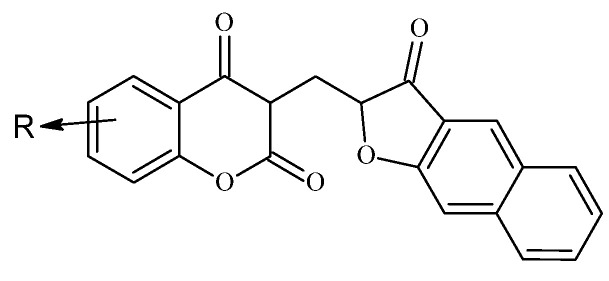
Chemical structures of compounds 23 series: **23a**: R = Me; **23b**: R = OMe.

A series of benzofuran–chromone–coumarin hybrids was synthesized and assessed against K562 leukemia cells, revealing robust cell-cycle perturbation and apoptosis consistent with therapeutic potential [75]. Structure–activity relationships (SAR) indicated that small substituent changes at the R positions markedly shift biological outcomes: the methyl analogue on the chromone ring (23a, R = Me) drove a pronounced G1 arrest (>70% of cells), whereas the methoxy counterpart (23b, R = OMe) predominantly caused S-phase blockade (~60%) Figure 30.

Halogen-substituted hybrids showed moderate pro-apoptotic activity, while introducing a naphthofuranone moiety substantially enhanced apoptosis and was associated with G2/M arrest, amplifying overall cytotoxicity versus parent scaffolds. Dose–response experiments corroborated these trends, indicating that subtle electronic/steric modifications tune phase specificity (G1, S, or G2/M), and that extended aromatic substitution elevates apoptotic potential. These findings position benzofuran–chromone–coumarin hybrids as versatile, tunable scaffolds for targeted leukemia therapy and provide a rational template for further optimization of potency and selectivity [76].

## 8. Coumarin-Based Derivatives for Targeted Stomach Cancer Therapy

Within gastric cancer (GC)—across AGS, MKN-45, SGC-7901, and HGC-27 models—coumarin scaffolds have been investigated as modulators of PI3K/Akt/mTOR and MAPK/ERK signaling, EGFR/HER2 and VEGFR-2–linked angiogenesis, with reproducible caspase-mediated apoptosis and G1/G2–M arrest; MDR/P-gp and context-dependent autophagy also emerge as modifiers of response and exposure. The subsections below highlight representative chemotypes and relate substitution patterns (e.g., C-4 aryl/benzyl, C-7 heteroaryl/alkoxy, linker polarity/length, halogenation) to these pathway-level outcomes.

For a concise map of motifs and targets pertinent to this section, see Section 9, Table 1.

### 8.1. Coumarin-Based Hybrids (SSBC, Compound **24**) as Pro-Apoptotic Agents in Gastric Carcinoma (AGS; BH3 Domain)

**Figure 31:**
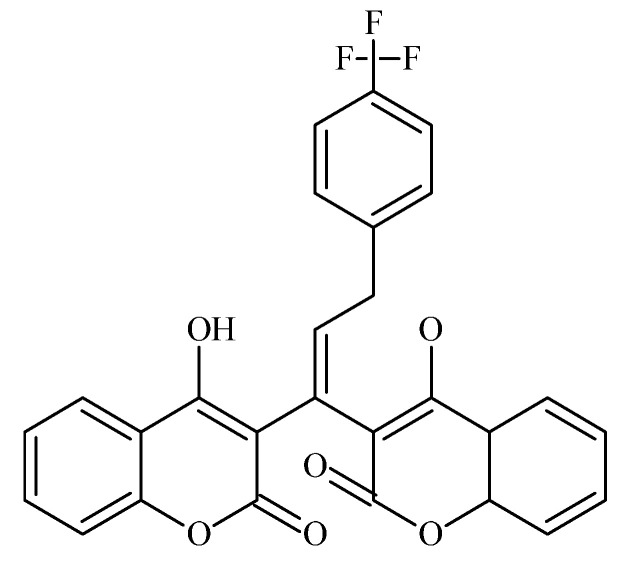
Chemical structure of compound **24**.

A synthetic coumarin-based hybrid designated SSBC (compound **24**, Figure 31) showed notable activity against AGS gastric-carcinoma cells, reducing viability in a dose-dependent fashion (apparent effects at 16–32 µg/mL) and suppressing colony formation; at concentrations >20 µg/mL, the compound displayed pronounced antiproliferative effects across assays [77].

Hallmarks of apoptosis were evident morphologically (nuclear condensation, membrane disruption by Hoechst and DAPI/PI staining) and by flow cytometry (annexin V–FITC/PI), which revealed substantial early- and late-apoptotic fractions. At the molecular level, treatment shifted the apoptotic balance toward the intrinsic (mitochondrial) pathway: BCL-2 decreased, while BAX, cytochrome c, and caspase-3 increased, with caspase-3 upregulation exceeding fourfold [77]. Docking supported engagement of the BH3 domain of anti-apoptotic targets, and ADME predictions indicated favorable pharmacokinetic properties. These data nominate compound **24** as a promising coumarin-based candidate for gastric cancer therapy, meriting further optimization and in vivo validation [78].

### 8.2. Coumarin and Furocoumarin Hybrids from Citrus trifoliata as Dual Antiproliferative and P-Glycoprotein Inhibitors in Colorectal Carcinoma (COLO 320; P-gp)

**Figure 32:**
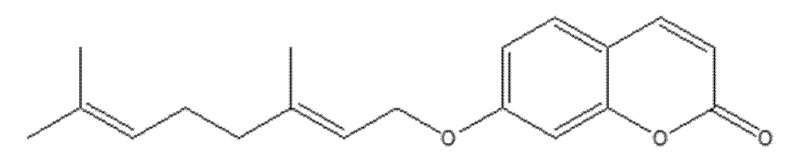
Chemical structure of compound **25**.

Coumarin-type metabolites isolated from *Citrus trifoliata* (Chinese/Japanese bitter orange) have drawn interest for anticancer applications. Recent phytochemical work identified ten constituents (the most active shown in Figure 32), including furocoumarins—imperatorin, phellopterin—and coumarins—scoparone (first reported for this species), myrsellin, triphasiol, umbelliferone, citropten—alongside limonoids.

In antiproliferative assays against COLO 320 colorectal adenocarcinoma cells, imperatorin and phellopterin showed moderate cytotoxicity, whereas citropten produced a more pronounced effect [16]. Significantly, phellopterin and citropten also inhibited P-glycoprotein (P-gp), reducing drug efflux and sensitizing cells to doxorubicin as verified by FACS, thereby combining direct growth suppression with chemosensitization. This dual action—antiproliferative activity plus MDR attenuation—underscores these scaffolds’ translational value and supports their consideration as adjuvants to standard chemotherapy. Overall, coumarin and furocoumarin derivatives from C. trifoliata warrant further mechanistic and in vivo evaluation to define selectivity, pharmacokinetics, and optimal combinations for colorectal cancer therapy [79].

### 8.3. 7-Diethylamino-4-Chloromethylcoumarin (7D4C) as a Selective Inducer of Mitochondria- and p53-Mediated Apoptosis in Colorectal Carcinoma (LoVo)

**Figure 33:**
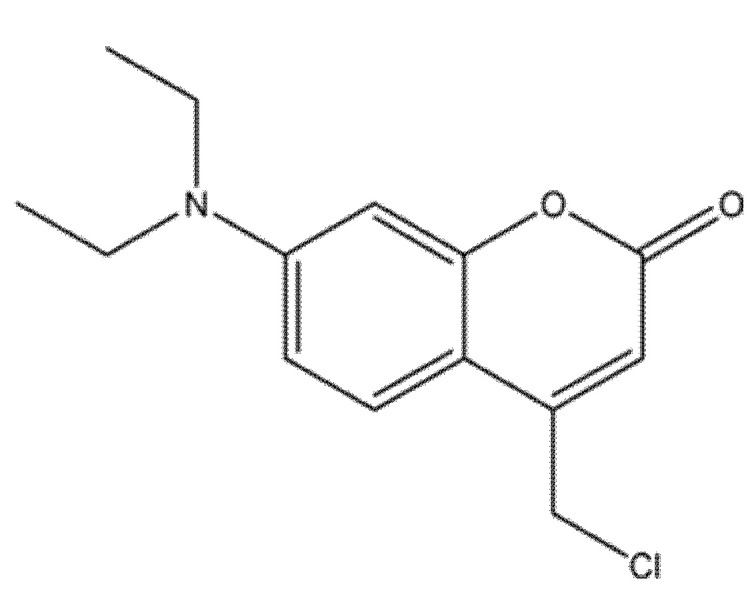
Chemical structure of compound **26**.

The novel coumarin derivative 7-diethylamino-4-chloromethylcoumarin (7D4C; compound **26** in Figure 33) was synthesized and structurally verified by FT-IR, ^1^H/^13^C NMR, and MALDI-TOF-MS, confirming purity and integrity. In comparative assays against LoVo colorectal adenocarcinoma cells and CCD-18Co normal fibroblasts, 7D4C showed selective cytotoxicity, markedly reducing LoVo viability while sparing non-malignant cells [80].

Mechanistic studies supported activation of the intrinsic (mitochondrial) apoptotic pathway: treatment disrupted mitochondrial membrane potential (MMP), elevated intracellular ROS and Ca^2+^, depleted GSH, and induced DNA strand breaks as evidenced by the comet assay. Molecular docking indicated strong binding to p53, suggesting that modulation of p53-dependent apoptotic signaling contributes to the observed effects. Although caspase activity was not directly quantified, the constellation of mitochondrial dysfunction and genotoxic stress is consistent with caspase-cascade engagement. Spectroscopic, computational, and biological data position 7D4C as a promising lead for colorectal cancer therapy; further dose–response refinement, caspase validation, pharmacokinetic profiling, and in vivo efficacy studies are warranted to establish translational potential [81].

### 8.4. Coumarin-Based Hybrids as Dual DNA-Polymerase Inhibitors and Antiproliferative Agents in Colorectal Cancer (HCT-116)

**Figure 34:**
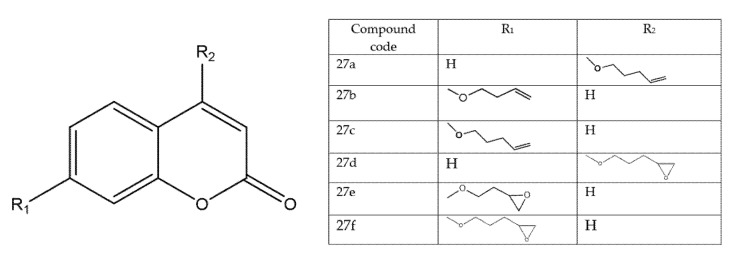
Chemical structures of compounds **27** series.

A structurally diverse library of coumarin-based hybrids was synthesized and profiled in complementary in vitro/in vivo assays (Figure 34). In enzyme studies, 27a and 27b inhibited Taq DNA polymerase with IC_50_ = 20.7 ± 2.10 μM and 48.25 ± 1.20 μM, respectively; molecular docking/dynamics indicated that 27a forms a stabilizing hydrogen bond with Glu345, whereas 27b engages Asp505, consistent with productive occupancy of the polymerase active site [82].

Notably, **27a** also contacted guanine and cytosine bases in modelled complexes, suggesting a dual mechanism that combines enzyme inhibition with direct DNA interaction [45]. In cell-based screens, **27c** emerged as the lead in colorectal cancer, selectively suppressing HCT-116 growth (IC_50_ = 8.47 μM; SI = 1.87). In parallel, epoxycoumarin analogues (**27d–27f**) produced DNA-damaging effects in Saccharomyces cerevisiae; **27f**—bearing a 4,5-epoxypentane substitution—was highlighted as a novel pharmacophore with promising activity. Beyond oncology, RT-PCR assays indicated antiretroviral potential for **27f** (IC_50_ = 134.22 ± 2.37 μM), attributed to its O-butylepoxy group, thereby extending the therapeutic scope of the scaffold. Collectively, these results position coumarin hybrids as multifunctional agents capable of targeting cancer-associated DNA polymerases while also exhibiting antiviral properties, and they motivate deeper mechanistic exploration, SAR optimization, and preclinical validation to translate this dual-action profile [76].

### 8.5. Ferrocene–Coumarin Hybrids as Selective Antiproliferative Agents in Colorectal Carcinoma (DLD-1)

**Figure 35:**
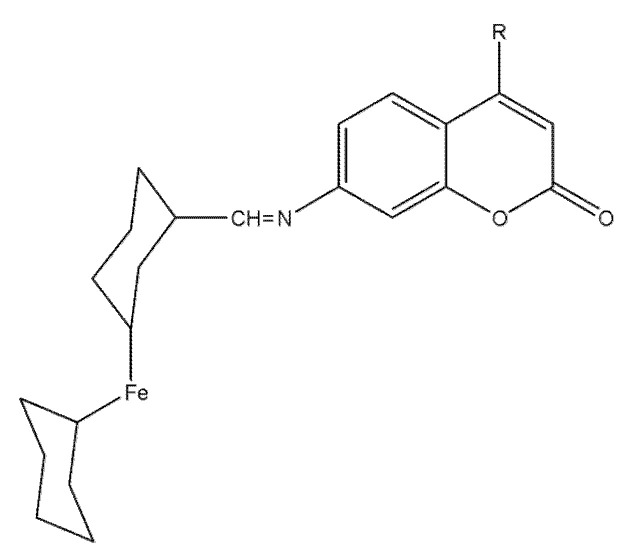
Chemical structure of compounds 28 series: **28a**: R = CH_3_; **28b**: R = CF_3_.

Two ferrocene–coumarin hybrids, 28a and 28b (Figure 35), were rationally designed to merge the coumarin pharmacophore with a redox-active ferrocenyl unit. Structures were confirmed by IR, UV–Vis, NMR, and MS, with diagnostic azomethine and ferrocenyl signals verifying successful synthesis and integrity.

In DLD-1 colorectal-carcinoma cells, both compounds reduced viability in a dose-dependent manner with IC_50_ = 64.3 µM (**28a**) and 67.1 µM (**28b**), while showing minimal toxicity toward normal colon epithelial cells—suggestive of a favorable therapeutic index and cancer-cell selectivity [83]. Fluorescence microscopy indicated efficient uptake with cytoplasmic and nuclear localization, consistent with access to critical intracellular targets; together with the intrinsic redox behavior of ferrocene, these observations implicate potential DNA engagement and redox-sensitive signaling effects. Overall, 28a/28b emerge as low-toxicity, selective leads whose structural versatility and intracellular distribution warrant SAR refinement, apoptotic-pathway validation, and in vivo assessment [84].

**Figure 36:**
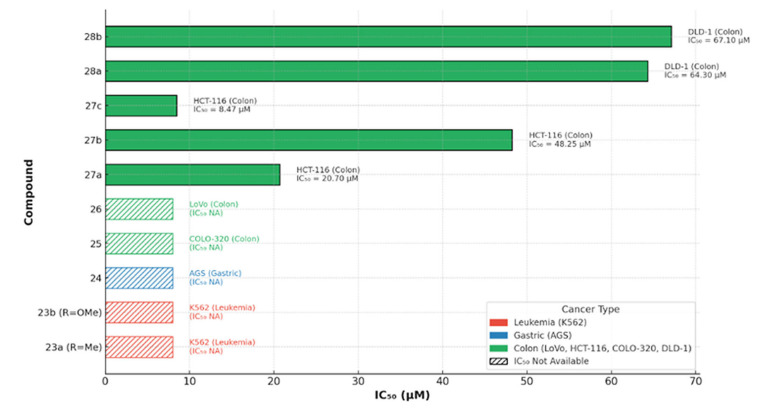
Cytotoxic activity of coumarin-based derivatives (23–28) against multiple cancer cell lines (K562, AGS, COLO-320, LoVo, HCT-116, DLD-1); comparison emphasizes the potency of 27-series analogues relative to 28a/28b.

To contextualize these results within this section’s broader panel, a cross-series comparison of coumarin derivatives **23–28** across hematologic and gastrointestinal models highlights transparent potency gradients (Figure 36): **27c** is the most active (IC_50_ = 8.47 µM, HCT-116), followed by **27a** (20.7 µM) and **27b** (48.25 µM); **28a/28b** are less potent in DLD-1 (both >60 µM), whereas **23a**, **23b**, **24**, **25**, and **26** show qualitative anticancer effects without reported IC_50_ values. These trends suggest that specific 27-series modifications markedly enhance colon-cancer cytotoxicity and could inform further optimization of the ferrocene–coumarin chemotype.

## 9. Structure–Activity Relationships and Recent Design Trends in Coumarin-Based Anticancer Agents

Coumarin-based anticancer agents display recurring, target-linked SAR motifs across breast, lung, prostate, and colorectal models. As summarized in Table 1, 4-aryl/benzyl, VEGFR-2–oriented coumarins deliver sub-micromolar kinase inhibition with G2/M-linked apoptosis (e.g., MCF-7 IC50 ≈ 1.24 µM; VEGFR-2 IC50 ≈ 0.36 µM), 7-substituted multitarget series engage EGFR/aromatase and often show higher potency in TNBC (MDA-MB-231) than ER-positive MCF-7, fluorinated scaffolds reinforce VEGFR-2/p38α interactions, and benzimidazole–coumarins attenuate PI3K/Akt/mTOR with caspase-dependent apoptosis. PARP-1/2–active coumarins reach nM enzyme potency with sub-µM cellular effects in BRCA-mutant lines, while P-gp–modulating coumarins act as chemosensitizers in MDR-high settings. Together, these patterns highlight actionable levers—C-4/C-7 substitution, linker length/polarity, and heteroaryl “warheads”—that tune pathway engagement and phenotypes (apoptosis, cell-cycle arrest) within biological context (ER+ vs. TNBC; autophagy-protective vs. non-protective). This table maps substitution motifs (e.g., C-4 aryl/benzyl, C-7 heteroaryl/alkoxy, linker polarity/length, halogenation) to primary targets and dominant phenotypes. The following Section 9.1, Section 9.2, Section 9.3, Section 9.4, Section 9.5, Section 9.6 and Section 9.7 organize these trends into target-focused themes and address resistance-aware design (MDR/autophagy) and translational constraints (ADME/PK/PD) to guide prioritization and combinations.

**Table 1:** Representative coumarin chemotypes: targets, models, potency/selectivity, and phenotypes.

Chemotype	Primary Target(s)	Model	Potency (IC50/Ki)	Dominant Phenotype	Citation
VEGFR-2-active coumarin (compound **4a**)	VEGFR-2 kinase	MCF-7	Cell IC50 1.24 µM; VEGFR-2 IC50 0.36 µM	G2/M arrest; caspase-9; pre-G1 apoptosis	[36]
7-substituted multitarget coumarins	EGFR; aromatase (CYP19A1)	MDA-MB-231 > MCF-7	MDA-MB-231 IC50 ≈ 1.9–3.5 µM; MCF-7 IC50 ≈ 3.9–6.0 µM	Apoptosis; G0/G1 or S arrest	[64]
Fluorinated coumarins (series)	VEGFR-2; p38α MAPK	MCF-7	VEGFR-2 inhibition up to 94%; cell IC50 7.9–8.3 µg/mL	Antiproliferative; kinase inhibition	[61]
Benzimidazole–coumarin hybrids	PI3K/Akt/mTOR axis	Breast-cancer models	Pathway suppression (biochemical)	Caspase-dependent apoptosis	[15]
Coumarin PARP-1 inhibitors (8-carbamyl-3-arylcoumarin)	PARP-1/2	BRCA-mutant lines (e.g., SUM149PT, HCC1937)	PARP-1 IC50 2.53 nM; PARP-2 IC50 6.45 nM; antiproliferative IC50 0.62–4.26 µM	G2/M arrest; ROS↑; DSB↑; apoptosis	[85]
P-gp (ABCB1) modulator LL-348 (coumarin)	MDR/P-gp chemosensitization	PK/transport models; combo with paclitaxel	Enhanced oral absorption and tumor uptake of paclitaxel	MDR reversal; chemosensitization	[86]
Aromatase-active coumarin (4-benzyl-3-(4′-chlorophenyl)-7-methoxy)	CYP19A1 (aromatase)	MCF-7aro	Ki ≈ 84 nM (competitive)	Suppresses androgen-driven proliferation (3D)	[87]
Hsp90-engaging coumarins (NTD/CTD concepts)	Hsp90 (NTD/CTD)	A549 ± in vivo	Cell IC50 (series-dependent)	Chaperone disruption; tumor-growth inhibition signals	[88]
Coumarin hybrids (Topo I/CDK2-oriented)	Topoisomerase I; CDK2	A549	Cell IC50 (series-dependent)	Cell-cycle arrest; apoptosis	[89,90]

Anchored to the patterns in the presented Table 1, we outline three feasible paths to individualize and potentiate coumarin-based strategies: align chemotypes with molecular typing—e.g., evaluate coumarin ± PARP inhibitor combinations in HRD/BRCA1/2 contexts [85,91]; pair PI3K/PTEN-altered tumors with coumarin designs that attenuate Akt/mTOR ± mTOR inhibitors [92,93]; in PD-L1–positive or otherwise immunogenic settings, test whether coumarin-driven apoptotic/angiogenic modulation can augment immune-checkpoint blockade [94,95]. In parallel, delivery-oriented optimization- such as tumor-targeted or long-circulating carriers to increase intratumoral exposure and reduce P-gp-mediated efflux—may enhance selectivity and efficacy [96]. Practical next steps include biomarker-stratified panels (ER+/TNBC; HRD; PI3K/PTEN), orthotopic/PDX models capturing MDR and autophagy phenotypes, and exposure–response PK/PD to confirm on-target activity in vivo.

### 9.1. VEGFR-2–Oriented Coumarins: 4-Aryl/Benzyl Patterns and G2/M-Linked Apoptosis

Coumarin cores bearing 4-aryl/benzyl motifs reproducibly engage VEGFR-2 and translate sub-micromolar kinase inhibition into G2/M-linked apoptosis in ER-positive models (e.g., MCF-7 IC50 ≈ 1.24 µM; VEGFR-2 IC50 ≈ 0.36 µM). Fluorinated variants can reinforce this profile via improved pocket interactions (p38α cross-talk observed in some series) [36,61].

### 9.2. EGFR/Aromatase-Biased Designs vs. Breast-Cancer Subtype (ER+ vs. TNBC)

7-Substituted, multitarget coumarins often co-engage EGFR and aromatase, with a recurrent potency shift favoring TNBC (MDA-MB-231) over ER-positive MCF-7; dedicated aromatase-active coumarins further support subtype-aware design and biomarker-guided selection [64,87].

### 9.3. PI3K/Akt/mTOR ± Autophagy: Protective vs. Non-Protective Settings

Reports of PI3K/Akt/mTOR attenuation by coumarins frequently coincide with autophagy induction, where autophagy is cytoprotective; coupling with autophagy blockers can unmask lethality [17]. Benzimidazole–coumarin chemotypes attenuate PI3K/Akt/mTOR signalling and elicit caspase-dependent apoptosis. Where autophagy proves cytoprotective, rational co-modulation (e.g., pairing with pathway or autophagy inhibitors) can convert partial responses into more durable cytotoxicity [15].

### 9.4. Hsp90 Engagement (NTD/CTD): Dual-Domain Strategies and In Vivo Signals

Hsp90-addressing coumarins can be framed within NTD/CTD dual-domain strategies; structural anchors such as NTD co-crystals provide a basis for pocket-compatible substitutions and for interpreting reported in-vivo tumor-growth-inhibition signals [88].

### 9.5. Topoisomerase I/CDK2 and Linker/Halogen SAR in A549

For Topo I/CDK2-oriented series, linker length/polarity and strategic halogen placement co-determine activity and the position of cell-cycle arrest in A549; structural references (CDK2; Topo I/DNA complexes) guide warhead/linker tuning [89,90].

### 9.6. MDR Chemosensitization (P-gp) and Delivery-Aware Optimization

Coumarins that inhibit P-gp can resensitize MDR models to doxorubicin, suggesting a rational pairing of signal-modulating scaffolds with chemosensitizers in P-gp-high tumors [16]. Moreover, coumarin modulators of P-gp (ABCB1) act as chemosensitizers that enhance intratumoral exposure to partner agents; integration with delivery-aware strategies (e.g., long-circulating or targeted systems) helps manage PK/DDI risks in MDR-high contexts [86].

### 9.7. Translational Design Rules: Potency ↔ Exposure ↔ Selectivity Funnel

Prioritization proceeds by a practical funnel: potency gate (enzyme/cell, phenotypes), exposure gate (ADME/PK; permeability/retention; P-gp if relevant), and selectivity gate (indices vs. non-malignant and subtype-matched panels). Candidates then enter mechanism-consistent combinations (e.g., PARP-i in BRCA-mutant settings) with biomarker-driven confirmation [85].

### 9.8. Cross-Indication Synthesis and Outlook

Across indications and chemical series surveyed in this review, coumarin scaffolds consistently modulate hallmark cancer pathways, yet do so with context-dependent efficacy. Mechanistically, the most recurrent axes include PI3K/Akt/mTOR (growth and survival), VEGFR-2 (angiogenesis), topoisomerase I/CDKs (DNA topology and cell-cycle control), EGFR/aromatase (receptor and steroid signaling), Hsp90 (proteostasis), and MDR/autophagy nodes that shape treatment response. This convergence is consistent with recent overviews of coumarins’ antitumor mechanisms and structure–activity trends, emphasizing how small substituent changes (e.g., halogens, heteroaryl linkers) can redirect target engagement and cellular phenotypes [5,11].

At the same time, biology–chemistry alignment proved critical: activity in one model did not always translate to another, even when nominal targets overlapped. For example, a selective VEGFR-2 inhibitor showed negligible cytotoxicity in PC-3 prostate cells in vitro, implying that VEGFR-2 signaling is not a dominant survival driver in that line despite strong enzyme-level potency [11,35,36]. Similar divergence appeared among A549-focused series, where linker length, halogen placement, or bulky hydrophobes altered potency, cell-cycle arrest points, and apoptotic readouts [9,38,39,40,41,42,43,44,45,53]. These observations are concordant with considerable pharmacogenomic efforts documenting discordant drug-response phenotypes across cell-line datasets and highlighting context dependence [97,98], as well as with evidence that genomic drift within nominally identical cell lines can alter drug sensitivity over time [99]. Accordingly, we recommend a development funnel that validates target dependence in the exact disease context, couples docking with orthogonal biochemical assays, and tests lead series across isogenic or phenotypically annotated models before animal work [100,101].

A recurring theme in the lung cancer sets was the bidirectional role of autophagy. In A549 cells, one coumarin–furoxan hybrid elicited non-protective autophagy that accompanied caspase-dependent apoptosis, whereas a related analogue induced protective autophagy that blunted cytotoxicity effects, as mirrored by pharmacologic or genetic blockade of autophagy in the primary studies [17,46,47,48,52]. Such context-contingent behaviour matches consensus reviews: autophagy can either sustain stressed tumor cells or potentiate cell death, and co-targeting (e.g., PI3K/mTOR ± autophagy inhibitors) is often required to convert ambiguous responses into durable killing [102,103,104].

In colorectal models, several citrus-derived coumarins and furocoumarins combined direct antiproliferative effects with P-glycoprotein (P-gp) inhibition, thereby re-sensitizing cells to doxorubicin [16,79]. This dual action is attractive given the central role of ABC transporters in clinical resistance; however, translation has often been limited by on-target transporter inhibition in standard barriers (intestine, liver, blood–brain barrier) and by pharmacokinetic drug–drug interactions. Contemporary reviews agree that effective P-gp modulation in oncology requires highly selective, low-liability inhibitors, repurposed agents with manageable interaction profiles, or context-specific delivery (rather than systemic, high-exposure blockade) [105,106,107]. The barrier role of P-gp at the BBB and other tissues underscores these constraints [108,109], and practical DDI frameworks further highlight intestinal/hepatic liabilities that should be addressed during development [110].

Among chaperone-targeted designs, the matrine–coumarin hybrid that dual-engages Hsp90 NTD and CTD achieved meaningful in vivo tumor growth inhibition with limited epithelial toxicity [49,50,51]. This aligns with the long-standing rationale for Hsp90 inhibition in oncology and current efforts to optimize domain selectivity, client degradation, and safety margins [111]. In particular, C-terminal domain (CTD) strategies—including inhibitors of the dimerization interface and CTD-biased chemotypes that avoid the heat-shock response typical of ATP-site NTD binders—have shown encouraging profiles [112,113,114], consistent with the tumor-selective Hsp90 conformations that underpin therapeutic windows [115]. These data suggest that polypharmacology within a validated chaperone system (e.g., simultaneous NTD and CTD engagement) may be more fruitful than piling disparate targets onto a single pharmacophore.

Collectively, the dataset highlights a design space that is chemically pliable but pharmacologically demanding. Several series showed good tumor–normal selectivity in vitro, yet comprehensive drug-likeness and exposure assessments were sparse. As coumarins often skew lipophilic with limited aqueous solubility, early, quantitative optimization of permeability/solubility (and liabilities such as time-dependent CYP inhibition) should be standard. Classic and still-relevant guidance (e.g., Rule-of-Five) and natural-product development playbooks emphasize the need to engineer ADME traits parallel with potency to avoid translation bottlenecks [116,117].

From a strategy perspective, three prioritization lanes emerge:

(1) Biomarker-guided triage—select models (and future patient subsets) with demonstrated dependence on the intended axis (e.g., VEGFR-2, PI3K/Akt, aromatase, CDK2/topo I, or Hsp90) before scaling chemistry; this aligns with best practice in biomarker-driven development and model selection 118,119,120.
(2) Resistance-aware combinations—pre-plan co-therapies to neutralize protective autophagy or efflux-mediated resistance, reserving systemic P-gp inhibition for delivery strategies that bias tumor exposure; consensus reviews highlight autophagy’s dual role and the translational challenges of ABC-transporter blockade [103,107,121].
(3) Translational pharmacology—iterate potency with PK/PD, metabolite, and tissue-distribution data (including PDX or immunocompetent models) to verify clinically reachable concentrations at the site of action [119,122,123].

**Figure 37:**
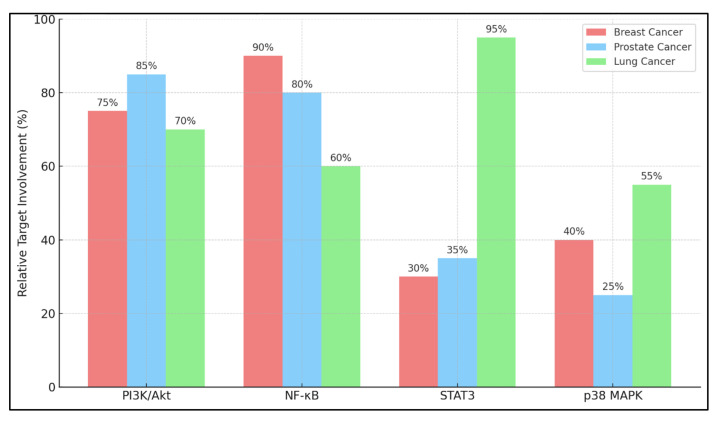
This review includes a cross-indication summary of signaling pathways targeted by coumarin derivatives. Relative involvement of PI3K/Akt, NF-κB, STAT3, and p38 MAPK across breast, prostate, and lung cancer models, based on studies summarized in Section 4, Section 5 and Section 6; symbol size reflects the number of reports; colors denote pathway class.

Figure 37 maps the distribution of pathway engagement by the coumarin chemotypes summarized in this review to synthesize the cross-indication patterns. As shown in Figure 37, breast- and prostate-oriented series cluster around VEGFR-2, aromatase/ER, and PI3K/Akt/mTOR modules, whereas lung entries more frequently engage topo I/CDK2, Hsp90, and p38 MAPK; autophagy and MDR nodes recur as modulators rather than primary drivers. These differences should guide model selection, biomarker strategy, and resistance-aware combinations in future studies.

Finally, we note that the strongest, most coherent preclinical signals in this review concentrate in ER-positive breast cancer (VEGFR-2/aromatase/ER modulation, caspase-9/BCL-2 axis) [61,62,63,64,65,66,67,68,69,70,78,79] and NSCLC (topo I/CDK2, Hsp90, PI3K/Akt/mTOR with autophagy as a modulatory node) [37,38,39,40,41,42,43,44,45,49,50,51,52,53]. In contrast, prostate-cancer results underscored the risk of target mismatch (e.g., VEGFR-2-centric designs in PC-3) and point to the need for biology-first candidate selection [11,35,36]. As the field advances coumarin frameworks with heteroaryl linkers, judicious halogenation, and solubility-enhancing modifications, the key to translation will be matching mechanism to dependence, confirming on-target engagement in vivo, and integrating safety/PK early rather than retrofitting these properties late in the pipeline [116,117,119,122].

## 10. Conclusions

Coumarin derivatives are adaptable small-molecule scaffolds with reproducible anticancer activity, modulating apoptosis, cell-cycle control, angiogenesis, and pro-survival signaling. Here we distill SAR motifs that link substitution patterns to pathway engagement and phenotypic outcomes. The strongest and most coherent signals arise in breast cancer—particularly ER-positive disease—where chemotypes engaging VEGFR-2/aromatase/ER and mitochondrial/caspase axes drive apoptosis and G2/M arrest; complementary signals are seen in lung models (topoisomerase I/CDK2, Hsp90, PI3K/Akt/mTOR, with autophagy as a modulatory node). Looking ahead, three priorities can accelerate translation: biomarker-stratified development aligning chemotypes with tumor biology (ER status, HRD/BRCA, PI3K/PTEN, MDR/autophagy phenotypes); mechanism-consistent combinations (PARP, mTOR or immune-checkpoint inhibitors) coupled to exposure–response confirmation of on-target action; and delivery- and exposure-focused optimisation to maximize intratumoral levels while managing efflux and DDI liabilities. With disciplined SAR, PK/PD, and orthotopic/PDX validation, coumarins are well-positioned to progress as selective, subtype-tailored oncology leads.

## Data Availability

The data presented in this study are available within this article.

## Abbreviations

The following abbreviations are used in this manuscript:

3-MA	3-Methyladenine (autophagy inhibitor)
ADME	Absorption, Distribution, Metabolism, Excretion
ADMET	Absorption, Distribution, Metabolism, Excretion, Toxicity
AEs	Adverse Events
AGS	Human gastric adenocarcinoma cell line
Akt	Protein kinase B
ALB/Alamar Blue	Resazurin-based cell viability assay
A549	Human lung adenocarcinoma cell line
ATG5	Autophagy-related 5
ATP	Adenosine triphosphate
BAX	Bcl-2–associated X protein
BCL-2	B-cell lymphoma 2
BEAS-2B	Human normal bronchial epithelial cell line
BBB	Blood–brain barrier
BH3	BCL-2 homology 3
CAR	Cytarabine
Casp-3/-9	Caspase-3/Caspase-9
CDK2	C-C motif chemokine 2
CC_50_	50% cytotoxic concentration
CDK CDK2	Cyclin-dependent kinase/Cyclin-dependent kinase 2
CTD	C-terminal domain (e.g., Hsp90-CTD)
DAPI	4′,6-Diamidino-2-phenylindole
DDI	Drug–drug interaction
DHFR	Dihydrofolate reductase
DLD-1	Human colon adenocarcinoma cell line
DMSO	Dimethyl sulfoxide
DOX	Doxorubicin
DU145	Human prostate carcinoma cell line
EGFR	Epidermal growth factor receptor
EMT	Epithelial–mesenchymal transition
ER/ERα	Estrogen receptor/Estrogen receptor-alpha
ERK1/2	Extracellular signal-regulated kinases 1/2
FACS	Fluorescence-activated cell sorting
FITC	Fluorescein isothiocyanate
FGFR	Fibroblast growth factor receptor
FT-IR	Fourier-transform infrared spectroscopy
GSH	Reduced glutathione
H1299/H460/Calu-1	Human NSCLC cell lines
HCT-116	Human colorectal carcinoma cell line
HEK-293	Human embryonic kidney cell line
HeLa	Human cervical carcinoma cell line
Hsp90	Heat shock protein 90
IC_50_	Half-maximal inhibitory concentration
iCa^2+^	Intracellular calcium
iROS	Intracellular reactive oxygen species
K562	Human chronic myelogenous leukemia cell line
LC3-II	Microtubule-associated proteins 1A/1B light chain 3B (lipidated form)
LoVo	Human colorectal adenocarcinoma cell line
LNCaP	Human androgen-responsive prostate cancer cell line
LY294002	PI3K inhibitor
MALDI-TOF-MS	Matrix-assisted laser desorption/ionization time-of-flight mass spectrometry
MAPK (p38α)	Mitogen-activated protein kinase (p38 alpha)
MCF-7	Human ER-positive breast cancer cell line
MCF-10A	Human normal breast epithelial cell line
MDA-MB-231	Human triple-negative breast cancer cell line
MDR	Multidrug resistance
MMP (ΔΨm)	Mitochondrial membrane potential
MST3	Mammalian Ste20-like protein kinase 3
MTT	3-(4,5-Dimethylthiazol-2-yl)-2,5-diphenyltetrazolium bromide assay
mTOR	Mechanistic target of rapamycin
NIH-3T3	Mouse embryonic fibroblast cell line
NMR	Nuclear magnetic resonance
NSCLC	Non-small-cell lung cancer
NTD	N-terminal domain (e.g., Hsp90-NTD)
PAINS	Pan-assay interference compounds
PARP	Poly(ADP-ribose) polymerase
PC-3	Human prostate carcinoma cell line
PD	Pharmacodynamics
PDB	Protein Data Bank
PDGFR	Platelet-derived growth factor receptor
PI (stain)	Propidium iodide
PI3K	Phosphoinositide 3-kinase
PK/PD	Pharmacokinetics/Pharmacodynamics
P-gp	P-glycoprotein (ABCB1)
PTEN	Phosphatase and tensin homolog
qRT-PCR	Quantitative real-time polymerase chain reaction
QSAR	Quantitative structure–activity relationship
RMSD	Root-mean-square deviation
ROS	Reactive oxygen species
SAR	Structure–activity relationship
SI	Selectivity index
SKOV-3	Human ovarian carcinoma cell line
TGI	Tumor growth inhibition
TNBC	Triple-negative breast cancer
TNF-α	Tumor necrosis factor-alpha
Topo I	Topoisomerase I
UV–Vis	Ultraviolet–visible spectroscopy
VEGFR-2	Vascular endothelial growth factor receptor-2
WI-38	Human lung fibroblast cell line
ΔΨm	Mitochondrial membrane potential
